# Amino acid substitution L232F in non-structural protein 6 identified as a possible human-adaptive mutation in clade B MERS coronaviruses

**DOI:** 10.1128/jvi.01369-23

**Published:** 2023-12-01

**Authors:** Ray T. Y. So, Daniel K. W. Chu, Kenrie P. Y. Hui, Chris K. P. Mok, Marcus H. H. Shum, Sumana Sanyal, John M. Nicholls, John C. W. Ho, Man-chun Cheung, Ka-chun Ng, Hin-Wo Yeung, Michael C. W. Chan, Leo L. M. Poon, Jincun Zhao, Tommy T. Y. Lam, Malik Peiris

**Affiliations:** 1 School of Public Health, Li Ka Shing Faculty of Medicine, The University of Hong Kong, Pokfulam, Hong Kong Special Administrative Region, People's Republic of China; 2 HKU-Pasteur Research Pole, School of Public Health, Li Ka Shing Faculty of Medicine, The University of Hong Kong, Hong Kong Special Administrative Region, People's Republic of China; 3 UK Health Security Agency, London, United Kingdom; 4 The Jockey Club School of Public Health and Primary Care, The Chinese University of Hong Kong, Hong Kong SAR, People's Republic of China; 5 Li Ka Shing Institute of Health Sciences, Faculty of Medicine, The Chinese University of Hong Kong, Hong Kong SAR, People's Republic of China; 6 Sir William Dunn School of Pathology, University of Oxford, Oxford, United Kingdom; 7 Department of Pathology, Li Ka Shing Faculty of Medicine, The University of Hong Kong, Pokfulam, Hong Kong Special Administrative Region, People's Republic of China; 8 State Key Laboratory of Respiratory Disease, National Clinical Research Center for Respiratory Disease, Guangzhou Institute of Respiratory Health, The First Affiliated Hospital of Guangzhou Medical University, Guangzhou, People's Republic of China; Emory University School of Medicine, Atlanta, Georgia, USA

**Keywords:** MERS-CoV, coronavirus, virus evolution, nsp6

## Abstract

Middle East respiratory syndrome coronavirus (MERS-CoV) causes zoonotic disease. Dromedary camels are the source of zoonotic infection. We performed sequence analysis of clade B MERS-CoV after excluding potential bias arising multiple sequences from a single zoonotic transmission chain and identified a substitution of amino acid leucine to phenylalanine in the codon 232 position of the non-structural protein 6 (nsp6) (nsp6 L232F) that occurs preferentially in human clade B MERS-CoV, with a rate of 16.9% (20/118) in human sequences vs a 0.6% (1/160) in camel sequences. Using a human clade B MERS-CoV strain GD01 as backbone, we generated a pair of isogenic recombinant MERS-CoV with nsp6 232L and 232F residues, respectively, and showed that the nsp6 L232F substitution confers higher replication competence in *ex vivo* culture of human nasal and bronchial tissues and in lungs of mice experimentally infected *in vivo*. Mechanistically, the nsp6 L232F substitution was found to associate with higher exocytic virus egress, while innate immune responses, autophagic restriction, and zippering activity of the endoplasmic reticulum remained unaffected. Our study suggests an adaptive mutation that occurs in clade B MERS-CoV associated with inter-species transmission to humans that may facilitate higher viral replication in the human respiratory tract. This highlights the importance of MERS-CoV as a zoonotic threat and the need for continued virus surveillance in camels and humans.

**IMPORTANCE:**

Viral host adaptation plays an important role in inter-species transmission of coronaviruses and influenza viruses. Multiple human-adaptive mutations have been identified in influenza viruses but not so far in MERS-CoV that circulates widely in dromedary camels in the Arabian Peninsula leading to zoonotic transmission. Here, we analyzed clade B MERS-CoV sequences and identified an amino acid substitution L232F in nsp6 that repeatedly occurs in human MERS-CoV. Using a loss-of-function reverse genetics approach, we found the nsp6 L232F conferred increased viral replication competence *in vitro*, in cultures of the upper human respiratory tract *ex vivo,* and in lungs of mice infected *in vivo*. Our results showed that nsp6 L232F may be an adaptive mutation associated with zoonotic transmission of MERS-CoV. This study highlighted the capacity of MERS-CoV to adapt to transmission to humans and also the need for continued surveillance of MERS-CoV in camels.

## INTRODUCTION

Middle East respiratory syndrome coronavirus (MERS-CoV) is an emerging pathogen that was first recognized in 2012 as a cause of severe acute respiratory diseases in humans (1). Although another novel coronavirus, severe acute respiratory coronavirus 2 (SARS-CoV-2) emerged in 2019 to cause a pandemic (COVID-19), MERS-CoV remains a potential pandemic threat and remains a research priority (2–4). As of December 2022, MERS-CoV had led to 2,603 confirmed human cases, 935 of which were fatal (5). Dromedary camels are the source of zoonotic human infection (6, 7). MERS-CoV-infected dromedary camels are found in the Arabian Peninsula (MERS-CoV clades A and B), in Africa (clade C), and Central Asia (phylogenetics undefined so far). Zoonotic disease has only been reported in the Arabian Peninsula (4, 8). The exact mode of inter-species transmission remains unknown, but entry via the respiratory or gastrointestinal tracts is the plausible transmission route and may occur via direct or indirect camel contact (8). There is no evidence of sustained transmission of MERS-CoV in the local community, although nosocomial and limited community outbreaks have occurred in Saudi Arabia (2014, 2015, 2016, and 2018) and South Korea (2015), which may sometimes exceed 100 or more individuals (9–12). Such human-to-human transmission occurred mainly between patients, healthcare workers, and visitors.

Genetic adaptation associated with inter-species transmission of viruses has been documented in SARS-CoV-1 and avian influenza virus (13, 14). SARS-CoV-2 also demonstrated genetic adaptation (e.g., D614G amino acid substitution in spike protein) associated with increased transmissibility and fitness within humans (15). Host adaptation mutations of MERS-CoV in humans remain poorly understood. Deletions in genes encoding accessory proteins have been reported, including one from a human MERS-CoV outbreak cluster and another one from an individual patient, but such variants occurred only on a single occasion (16, 17). It is important to investigate host-adaptive mutations associated with zoonotic transmission of MERS-CoV.

In this study, we aimed to identify mutations that were biased to occur more frequently in human rather than in dromedary camel MERS-CoV sequences. We adjusted the genetic diversity differences between the camel and human MERS-CoV sequence data set and identified an nsp6 L232F substitution that preferentially occurred in human MERS-CoV sequences. Using reverse genetics, we demonstrated that the mutation conferred higher replication competence in the human respiratory tract. MERS-CoV nsp6 was previously shown to interact with different cellular processes, in particular, autophagy restriction and innate immune antagonism (18–20). Nsp6 has also recently been shown to play an important role in the biogenesis of viral-induced double-membrane vesicles (DMVs) and is known to be a replication organelle for viral RNA synthesis through zippering of endoplasmic reticulum (ER) (21). We could not demonstrate that the mutation induced significant changes in autophagy and ER zippering during infection. Our study highlighted the need for a continued surveillance of MERS-CoV evolution.

## RESULTS

### Repeated independent emergence of nsp6-232F in multiple lineages of human MERS-CoV isolates suggests convergent evolution

**Fig 1:**
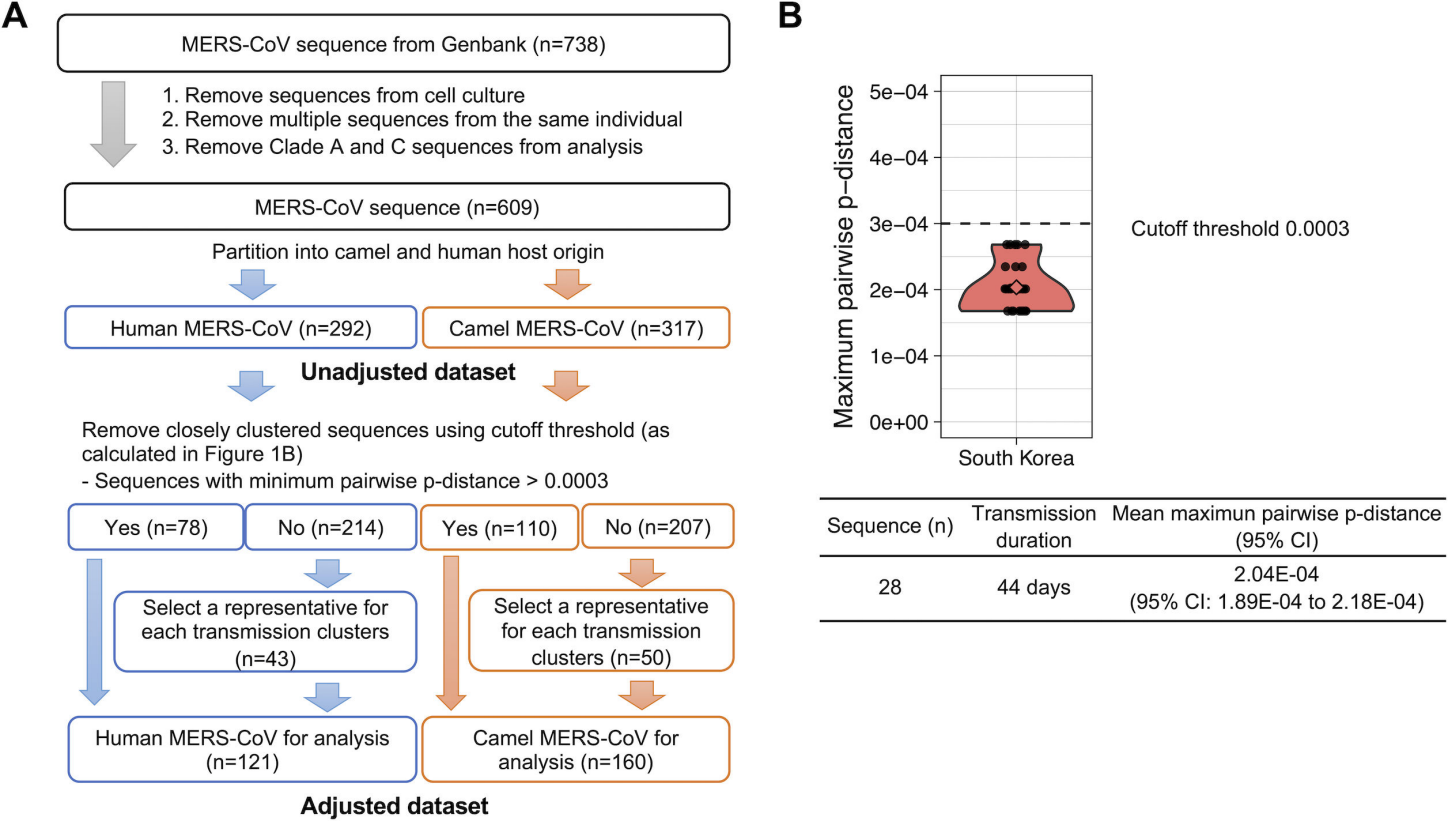
Data set preparation for identification of human-adaptive mutations in MERS-CoV. (**A**) Scheme of data set adjustment for sequence analysis to avoid bias in the sequence sources. A total of 738 MERS-CoV complete and partial (>10 kb) genomes from camel and human were downloaded from GenBank. To avoid bias or oversampling of case clusters, sequences derived from cell culture and multiple sequences from the same individual were removed. Clade A (no camel sequences) and C (no human sequences) virus sequences were also removed. Finally, a threshold was applied to exclude highly similar sequences that potentially may arise from a single zoonotic event. Thus, 121 human and 160 camel MERS-CoV were included for mutation analysis. (**B**) Diversity of MERS-CoV genomes in the South Korea outbreak. A violin plot showing the maximum pairwise *p*-distances for each sequence (refer to Materials and Methods) within the outbreak. *p*-Distance is calculated using SSE (v1.4). Maximum pairwise *p*-distance of 0.0003 was used for data set trimming.

We retrieved a total of 738 MERS-CoV complete and partial (>10 kb) genomes from GenBank (NCBI, NIH) to identify mutations that were preferentially biased to occur in human MERS-CoV. Since there are substantial sampling differences between MERS-CoV from humans and camels, in which human MERS-CoVs were mainly sampled at high intensities during hospital outbreaks, whereas camel MERS-CoV were mostly sampled from active surveillance in camel herds, we attempted to adjust this sampling bias by removing those highly similar, oversampled human MERS-CoV sequences. The overall scheme of our filtering approach included removal of sequences derived from cell culture and serial sampling of the same patient, removal of clade A sequences (clade A has no corresponding camel sequences for comparison) and clade C sequences (which have not so far resulted in documented zoonotic events), and removal of closely clustered or genetically identical MERS-CoV sequences (Fig. 1A, refer to Material and Methods for details). Since we wanted to exclude bias potentially arising from including multiple sequences from a single zoonotic transmission chain, we used the mutation distance observed within sequences of the outbreak in South Korea in 2015 as a reference. This outbreak represented one of the largest and best documented chains of human-to-human transmission documented so far. We utilized a minimum pairwise *p*-distance of 3e−4, well above the distance observed in the South Korean outbreak, as our threshold cutoff to define genetically distinct sequences to be added for analysis (Fig. 1B). For sequences beyond the threshold, one representative sequence from each different genetic cluster was added to the final data set for analysis (details refer to Materials and Methods). The same cutoff threshold was applied to remove closely clustered camel sequences. Using the adjusted data set with 121 human sequences and 160 camel sequences, we first performed sequence-based analysis and screened amino acid substitutions with higher rates in humans than camels as potential adaptive mutations in human MERS-CoV. Using these criteria, the mutation with the greatest significance upon Fisher’s exact tests was the nsp6 L232F substitution, which occurred in 20/118 (16.9%) of the human sequences but in just 1/160 (0.6%) of the camel MERS-CoV sequences (Table 1A). It was also the only hit showing a statistical difference after adjusting for multiple tests using Bonferroni correction. In parallel, the nsp6 L232F substitution was also the greatest significance hit in the unadjusted data set (Table 1B).

**TABLE 1:** List of potential human-adaptive mutations in MERS-CoV clade B^*a*^

A) Adjusted data set		Sequence analysis						Phylogenetic analysis		
Protein	Substitution	Human MERS-CoV (*n* = 121)		Camel MERS-CoV (*n* = 160)		Rate difference	Fisher's exact test *P*-value	Model likelihood of host independent evolution	Model likelihood of host dependent evolution	Log Bayes factor
		Rate	n	Rate	n					
nsp6	L232F	16.9%	(20/118)	0.6%	(1/160)	16%	2.2E-07^*b*^	−294.46	−292.19	4.54^*c*^
ORF3	P86L	63.7%	(72/113)	39.4%	(61/155)	24%	1.17E-04	−251.59	−261.30	−19.42
ORF4b	M6T	60.2%	(71/118)	37.7%	(60/159)	22%	0.00026	−279.06	−291.13	−24.14
N	A300V	7.8%	(9/115)	0.0%	(0/160)	8%	0.00032	−237.40	−234.79	5.22^*c*^
nsp3	A609T	21.8%	(26/119)	7.5%	(12/160)	14%	0.00071	−224.71	−232.00	−14.57
nsp3	P246R	23.1%	(27/117)	8.8%	(14/160)	14%	0.00110	−228.59	−236.16	−15.15
M	F123I	65.5%	(76/116)	46.5%	(74/159)	19%	0.00215	−244.25	−255.44	−22.40
nsp13	V241A	5.8%	(7/120)	0.0%	(0/160)	6%	0.00240	−252.97	−250.06	5.81^*c*^
nsp3	G128S	22.7%	(27/119)	9.4%	(15/160)	13%	0.00361	−229.34	−237.67	−16.65
nsp3	V522I	22.7%	(27/119)	9.4%	(15/160)	13%	0.00361	−234.12	−241.51	−14.79

B) Unadjusted data set		Sequence analysis						Phylogenetic analysis		
**Protein**	**Substitution**	**Human MERS-CoV (*****n*** **= 292)**		**Camel MERS-CoV (*****n*** **= 317)**		**Rate difference**	**Fisher's exact test *P*-value**	**Model likelihood of host independent evolution**	**Model likelihood of host dependent evolution**	**Log Bayes factor**
		**Rate**	**n**	**Rate**	**n**					
nsp6	L232F	28.0%	(80/286)	0.3%	(1/317)	28%	2.20E-16^*b*^	−330.65	−327.31	6.69^*c*^
N	L283F	11.2%	(31/277)	0.0%	(0/315)	11%	2.32E-11^*b*^	−232.98	−232.08	1.81
N	S11F	11.1%	(31/280)	0.0%	(0/316)	11%	2.66E-11^*b*^	−232.95	−231.98	1.94
nsp3	D124G	10.9%	(31/285)	0.0%	(0/314)	11%	4.05E-11^*b*^	−232.45	−231.89	1.11
S	I529T	7.1%	(20/282)	0.0%	(0/316)	7%	2.05E-07^*b*^	−284.98	−284.14	1.68
nsp3	A176V	6.8%	(19/278)	0.0%	(0/314)	7%	4.13E-07^*b*^	−231.15	−230.81	0.68
S	S262A	6.8%	(19/281)	0.0%	(0/317)	7%	4.20E-07^*b*^	−283.36	−283.14	0.45
nsp3	S171F	6.7%	(19/283)	0.0%	(0/314)	7%	4.99E-07^*b*^	−231.19	−230.73	0.91
nsp3	V270I	6.7%	(19/284)	0.0%	(0/314)	7%	5.17E-07^*b*^	−231.18	−230.69	0.98
nsp3	T562N	6.7%	(19/285)	0.0%	(0/314)	7%	5.37E-07^*b*^	−231.19	−231.05	0.29

^*a*^ List of top 10 hits of human-host biased mutations in (A) adjusted and (B) unadjusted MERS-CoV data set. Hits were sorted by *P*-value significance. Each amino acid substitution between human and camel MERS-CoV was compared for independence using Fisher’s exact test. Only mutations with rates higher in humans than camels were included. Mutation hits were tested for correlated evolution between pairs of host and mutation traits using BayesTraits (v4.0.0). Log Bayes factors of likelihood between the discrete dependent and independent models were shown.

^*b*^ Mutations with statistical significance using Fisher’s exact test. A *P*-value <6.93E−5 for adjusted data set and a *P*-value <5.19E−5 for unadjusted data set considered as significant after Bonferroni correction for multiple testing.

^*c*^ Mutations with positive evidence for correlated evolution between the host trait and mutation trait. <2 indicates weak evidence, ≥2 indicates positive evidence, and 5−10 indicates strong evidence.

**Fig 2:**
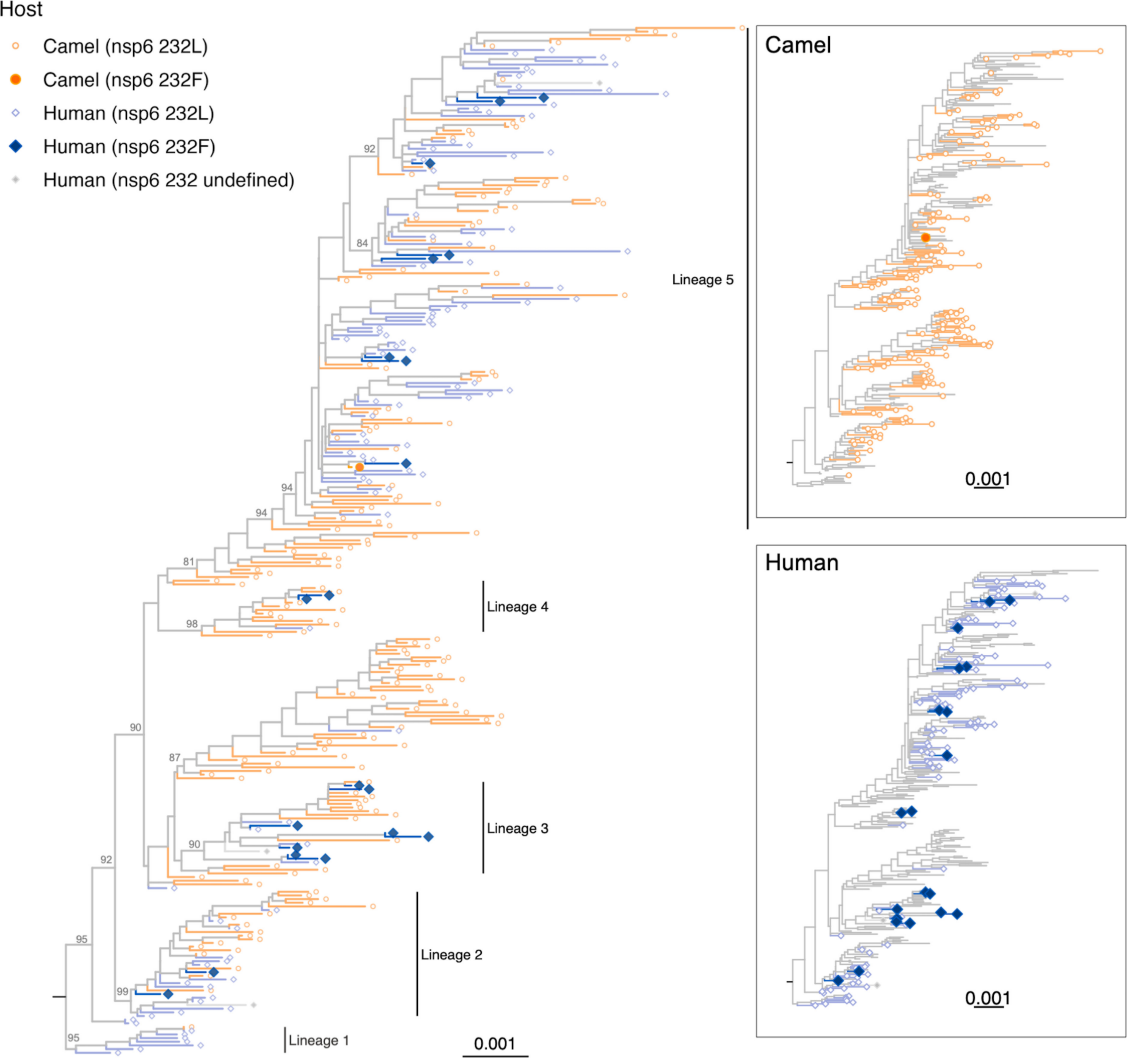
The occurrence of MERS-CoV nsp6 L232F in human and dromedary camel clade B MERS-CoV. A maximum likelihood tree of 281 clade B MERS-CoV nucleotide sequences labeled with the host and the presence of the mutation. Insets labeled with only camel or human sequences were shown on the right. The tree was built with IQ-Tree v1.6.12 using GTR + I + R substitution model selected by model testing. Yellow circles represent camel sequences; blue diamonds represent human sequences. A taxon with larger filled color shape indicates the presence of the nsp6 L232F substitution. Three sequences (human nsp6 232 undefined) without the nsp6 codon 232 information are indicated as filled gray diamonds in the taxon. Scale bar, 0.001 nucleotide change per genomic position. Deep internal nodes with bootstraps >80 are indicated.

We further employed phylogenetic methods to test whether the identified nsp6 L232F substitution is supported as host-dependent evolution using BayesTraits (22). Model likelihood tests showed nsp6 L232F, N A300V, and nsp13 V241A have positive evidence (log Bayes factors >2) to support dependent evolution of these mutation traits with host traits, indicating these mutations were more associated to occur in humans (Table 1A). Similarly, positive evidence of host-dependent evolution was also observed in the unadjusted data set for the nsp6 L232F substitution (Table 1B). The nsp6 L232F hit showed no strong correlation with other hits as epistatic effects (phi coefficient <0.3) (Fig. S1). A phylogenetic tree showed the nsp6 mutation emerged independently in multiple lineages of MERS-CoV (clade B lineages 2, 3, 4 and 5) associated with zoonotic transmission, suggesting convergent evolution of this mutation in clade B MERS-CoV (Fig. 2, enlarged version in Fig. S2). A phylogenetic tree with the unadjusted data set including closely related sequences is shown for comparison (Fig. S6).

### Isogenic virus clones generated by reverse genetics showed that MERS-CoV nsp6-232F is associated with enhanced replication in Calu-3 cells, Vero cells, and hDPP4 knockin mice

**Fig 3:**
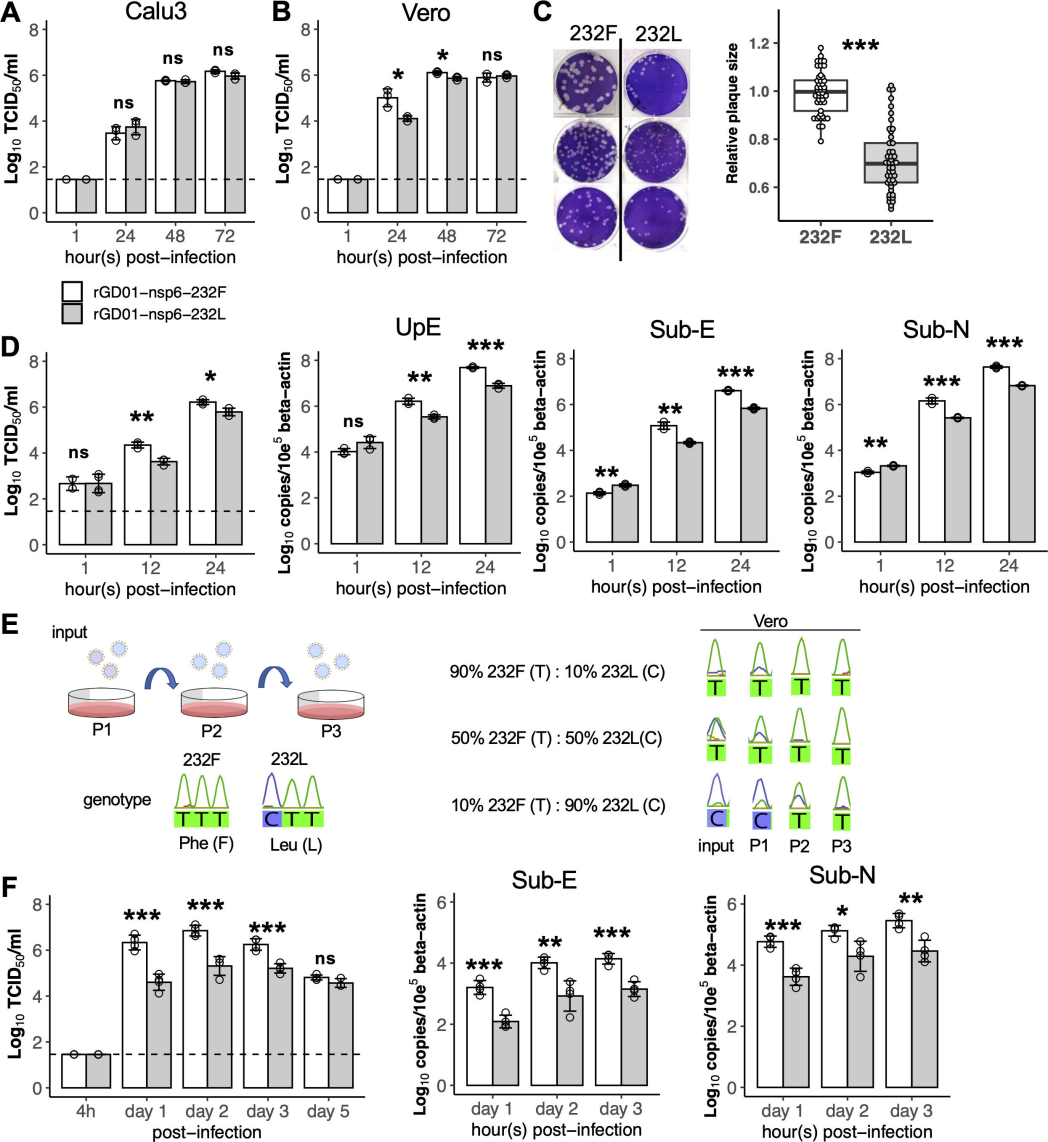
Comparison of virus replication kinetics of rGD01-nsp6-232F and rGD01-nsp6-232L isogenic MERS-CoV. (**A**) Calu-3 cell cultures and (**B**) Vero cell cultures were infected at an MOI of 0.01, and virus titers in culture supernatants were determined by TCID_50_ assay. Assays were performed in three independent experiments with triplicates in each. Dots represent mean  ±  s.d. for each experiment. (**C**) Relative plaque sizes of rGD01-nsp6-232F and rGD01-nsp6-232L virus were determined in Vero cells. A total of *n* = 46 plaques were analyzed for each virus. The box plot displays median and interquartile range. Performed in three independent experiments. (**D**) Early cycles replication kinetics in Vero cells (*n* = 3) at MOI = 2 measured for live virus production and RNA synthesis. Assays were performed in triplicates. Data are mean  ±  s.d. (**E**) Schematic setup of virus growth competition assay in Vero cells. Vero cells were infected with different ratios (9:1, 1:1, and 1:9) (*n* = 1) of rGD01-nsp6-232F and rGD01-nsp6-232L virus and serially passaged three times. Genotype of the nsp6 amino acid 232 position was determined by Sanger sequencing. (**F**) Viral titers in the lungs of human DPP4-knockin mice infected intranasally with 1 × 10^4^ plaque-forming units of each virus (*n* = 4 for each group). Lung homogenates (*n* = 4) were measured for viral titers by TCID_50_ assay, and total RNA was extracted to measure subgenomic viral RNA templates by RT-qPCR targeting the envelope (subgenomic E) and nucleocapsid (subgenomic N) gene. Statistical tests were done using two-tailed Student’s *t* test: *P* ≥ 0.05 (ns); *P* < 0.05 (*); *P* < 0.01 (**); *P* < 0.001 (***).

To study the effect of the nsp6 L232F substitution on viral replication, we used reverse genetics to rescue a pair of isogenic recombinant viruses from a wild-type human clade B MERS-CoV strain, ChinaGD01, that contains the nsp6-232Phe mutation (rGD01-nsp6-232F) and a hypothesized loss-of-function isogenic mutant of ChinaGD01 that was mutated to carry the camel predominant Leu residue in the nsp6 232 position (rGD01-nsp6-232L). In Calu-3 cells, both rGD01-nsp6-232F and rGD01-nsp6-232L viruses replicated similarly in multicycle infections [multiplicity of infection (MOI) = 0.01] from 24 to 72 hours post-infection (hpi) (Fig. 3A). In Vero cells, that are deficient in type I interferon (IFN-I) production, rGD01-nsp6-232F replicated to significantly higher viral titers than rGD01-nsp6-232L at 24 and 48 hpi (MOI of 0.01) from three independent experiments (Fig. 3B). Plaque morphology in Vero cells showed that rGD01-nsp6-232F formed plaques of larger sizes than rGD01-nsp6-232L (fold difference: 1 vs 0.71, three independent experiments) (Fig. 3C). The pair of viruses were infected at an MOI of 2 to measure early viral kinetics, and again rGD01-nsp6-232F showed higher viral replication in infectious virus assays as well as higher levels of UpE targeting genomic copies and subgenomic viral RNA synthesis at 12 and 24 hpi in Vero cells (Fig. 3D). A direct growth competition assay between the pair of isogenic viruses was performed in Vero cells with different ratios of rGD01-nsp6-232F and rGD01-nsp6-232L ranging from 9:1, 1:1, and 1:9, at an MOI of 0.01 (Fig. 3E). After three rounds of serial passage, nsp6 232Phe emerged as the dominant genotype at all infection ratios in Vero cells, supporting the contention that rGD01-nsp6-232F has higher intrinsic fitness and replication competence.

Viral kinetics were then compared in the human DPP4 knockin C57BL/6 mouse model that has been previously used for MERS-CoV replication studies (23). Following intranasal inoculation of 10^4^ plaque-forming units (pfu) of each isogenic virus per mouse, lung viral titers were significantly higher in rGD01-nsp6-232F virus at 1 to 3 days post-infection (dpi), with the greatest difference of 1.71 log_10_ 50% tissue culture infection dose (TCID_50_)/mL observed on day 1 (Fig. 3F). Titer differences between the viruses gradually diminished from day 2 to day 5. Higher subgenomic RNA production was seen in rGD01-nsp6-232F than in rGD01-nsp6-232L from day 1 to 3 dpi. Overall, these data suggest the nsp6 L232F substitution conferred greater replication competence in the mouse respiratory tract.

### MERS-CoV nsp6-232F exhibits enhanced replication in *ex vivo* cultures of the human upper and lower respiratory tract

**Fig 4:**
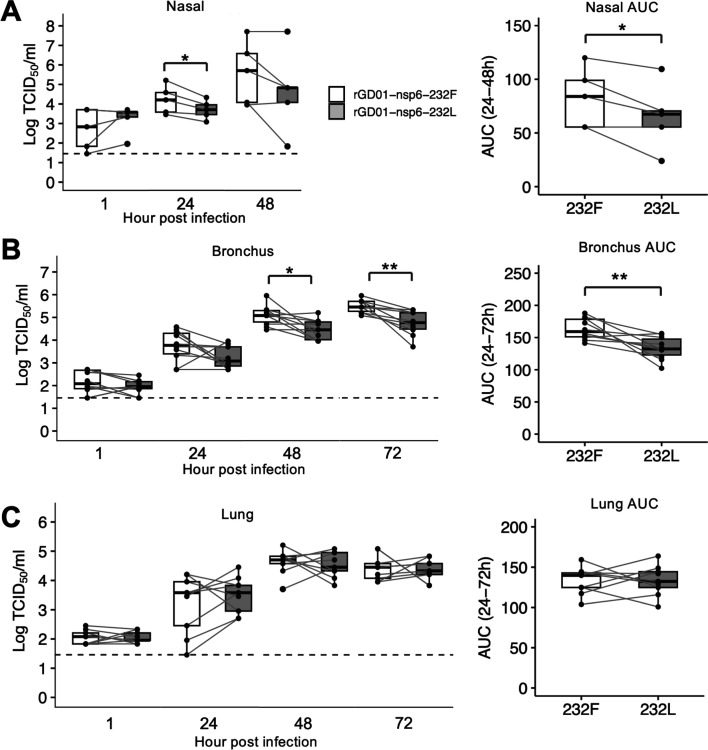
Comparison of virus replication kinetics in *ex vivo* cultures of nasal, bronchial, and lung tissues. *Ex vivo* cultures of nasal (**A**), bronchus (**B**), and lung (**C**) tissues were infected with a dose of 10^6^ TCID_50_ of rGD01-nsp6-232F and rGD01-nsp6-232L viruses. Virus titers in culture supernatants at the indicated timepoints were determined by TCID_50_ assay. Data were generated from three individual tissue donors for nasal and five donors for bronchus and lung. Data from different timepoints of the same tissue donor were connected with horizontal lines. The horizontal dotted line denotes the limit of detection in the TCID_50_ assay. Statistical tests were done using paired *t* test: *P* < 0.05 (*), *P* < 0.01(**).

To evaluate viral replication competence and potential changes of tropism in the human respiratory tract, we assessed the effect of the nsp6 L232F substitution in *ex vivo* cultures of nasal, bronchial, and lung tissues from at least three human donors. We compared the viral replication kinetics of the isogenic viruses at 33°C in nasal turbinate tissues and at 37°C in bronchus and lung tissues. In the nasal tissues, rGD01-nsp6-232F showed significantly higher virus titer at 24 hpi, but titers were not significantly different at 48 hpi. Aggregating virus titers at 24–48 hpi using area under the curve (AUC) analysis showed an overall increase in the release of infectious virus in nasal tissues (Fig. 4A). Similarly, rGD01-nsp6-232F showed significantly higher virus titers at 48 to 72 hpi, as well as in the AUC analysis, in *ex vivo* cultures of human bronchus (Fig. 4B). No significant differences in virus titers were observed in lung tissues at 24, 48, or 72 hpi (Fig. 4C). Overall, the nsp6 L232F showed enhanced viral replication in human upper respiratory tract and conducting airway tissues.

### MERS-CoV nsp6-232F does not affect innate immune antagonism in Calu-3 cells

**Fig 5:**
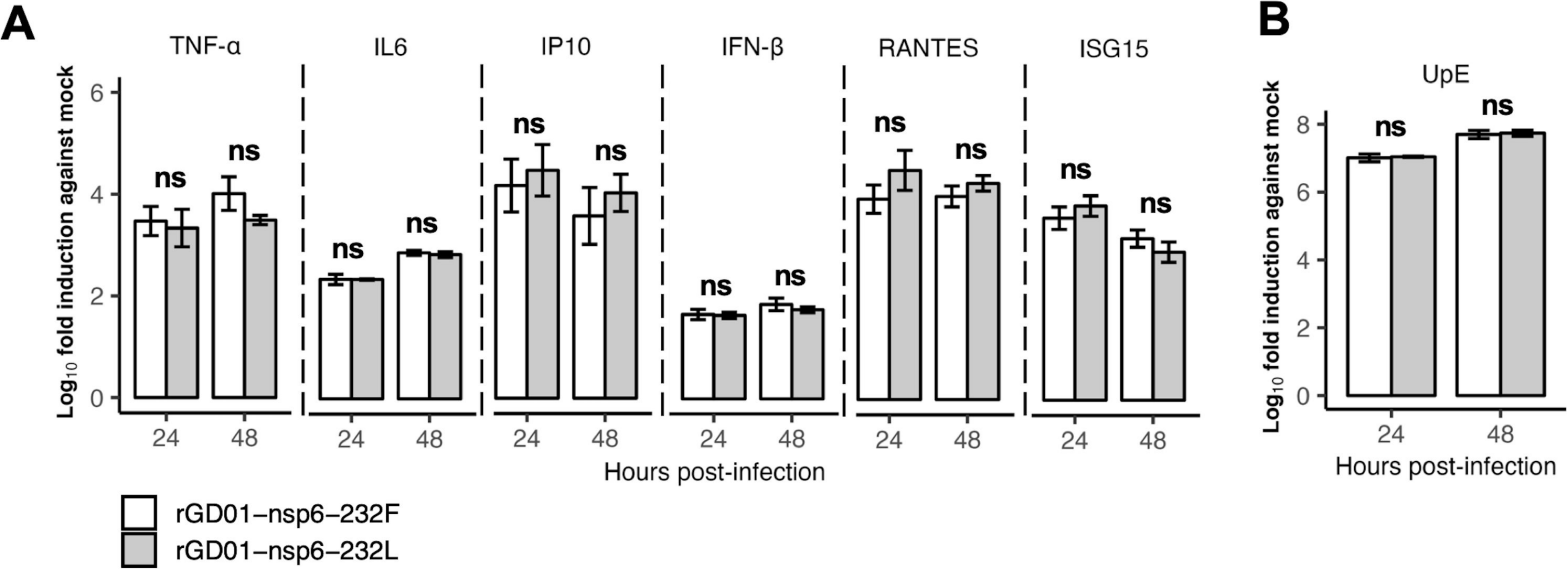
Innate immune gene expression in Calu-3 cells infected with rGD01-nsp6-232F and rGD01-nsp6-232L viruses (MOI = 2). (**A**) mRNA expression of immune genes (TNF-α, IL6, IP10, IFN-β, RANTES, and ISG15) was determined using qPCR from cells infected with rGD01-nsp6-232F or rGD01-nsp6-232L virus. Mock control was cells mock infected without virus infection. Assays were performed in triplicates. Data are mean  ±  s.d. (**B**) Quantification of MERS-CoV RNA copies using RT-qPCR targeting the upstream region of E gene (UpE). Assays were performed in biological triplicates. Data are mean  ±  s.d. Statistical tests were done using the Student’s *t* test: *P* ≥ 0.05, not significant (ns).

To delineate the effect of nsp6 L232F substitution on innate immune responses and address whether the replication phenotype in Calu-3 cells was masked by altered innate immune antagonism, we tested innate immune responses elicited by infection of Calu-3 cells with the pair of isogenic viruses. Nsp6 has previously been shown to inhibit IFN-I production *in vitro* in an overexpression system (20). We measured the mRNA expression of a panel of IFN-stimulated genes in Calu-3 cells, at 24 and 48 hpi following infection with each virus at an MOI = 2. No significant differences in the mRNA expression of IFN-β, TNF-α, IL6, ISG15, RANTES, and CXCL-19 (IP-10) were observed at both 24 and 48 hpi (Fig. 5A), with similar levels of viral RNA copies (Fig. 5B). This suggests nsp6 L232F substitution did not alter innate immune antagonism upon infection.

### MERS-CoV nsp6-232F showed minimal modulation in both autophagy in Vero cells and zippering of endoplasmic reticulum in Hela cells

**Fig 6:**
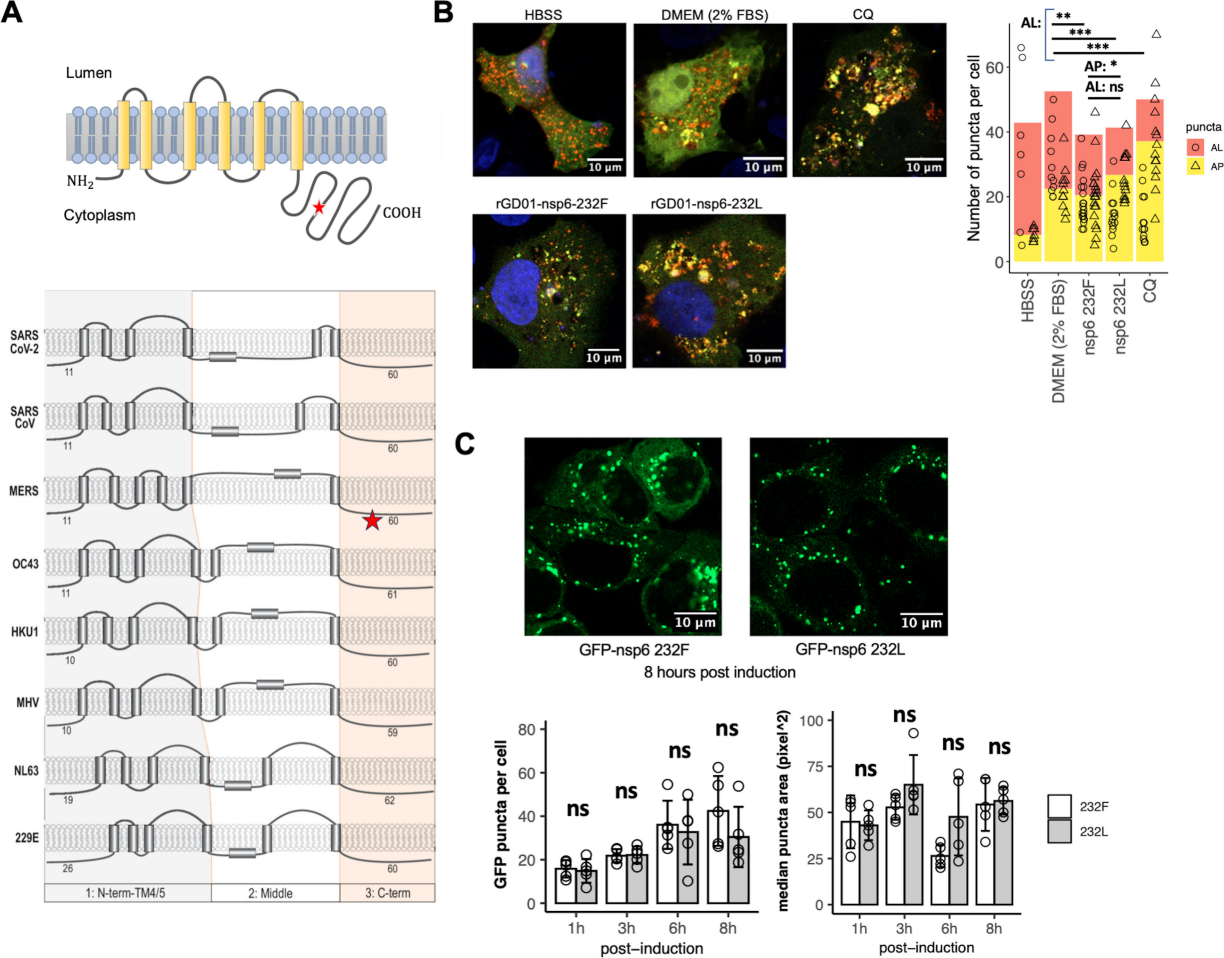
Nsp6 L232F substitution showed minimal modulation in autophagy and ER zippering. (**A**) Illustration of the topology of MERS-CoV based on a previous study of murine coronavirus nsp6 from Baliji et al. (24) and the topology of nsp6 in multiple coronaviruses from Feng et al. (25). The conserved domain is shaded orange as from the original illustration. The position of the L232F mutation is indicated as the star icon. (**B**) Vero cells transfected with mCherry- and EGFP-tagged LC3 were infected with each of the recombinant viruses at MOI = 0.01 and processed for immunofluorescence at 24 hpi. Hanks’ balanced salt solution (HBSS) induced cell starvation and was used as a high autophagic flux control. Dulbecco’s modified Eagle medium (DMEM) supplemented with 2% fetal bovine serum (FBS) acts as the basal autophagic flux control. Chloroquine (CQ, 100 mM) inhibits AP fusion with AL as low autophagic flux control. Images are representative of three biological replicates. (**B**) Summary statistics of the number of AP and AL puncta counted. Number of cells analyzed: HBSS (*n* = 7); DMEM 2% FBS (*n* = 11); WT (*n* = 20); nsp6 232L (*n* = 14). (**C**) Analysis of ER-zippering formation in Hela cells expression GFP-nsp6 WT or GFP-nsp6 232L induced by doxycycline. GFP puncta per cell and area of puncta were measured at 1, 3, 6, and 8 hours post induction. Images were analyzed using ImageJ. Each setup was analyzed with a cell number ranged of *n* = 35–59. Images are representatives of three biological replicates. Statistical tests were done using two-tailed Student’s *t* test: *P* ≥ 0.05 (ns); *P* < 0.05 (*); *P* < 0.01 (**); *P* < 0.001 (***).

The nsp6 L232F substitution is expected to be located at the conserved protein c-terminal cytoplasmic domain (Fig. 6A). CoV nsp6 has been shown to involve autophagy restriction and biogenesis of double-membrane vesicles through zippering of ER (18, 21). We studied the effect of the nsp6 L232F substitution on the induction and flux of autophagy in Vero cells, using a tandem fluorescent-tagged LC3 (mRFP-EGFP-LC3) which can visualize autophagic compartments [red puncta = autolysosomes (AL); yellow puncta = autophagosomes (AP)]. Infection of both viruses at MOI = 0.01 reduced AL puncta, when compared to mock control, suggesting restriction of autophagy from both viruses (Fig. 6B). A slight reduction of AP puncta was observed in rGD01-nsp6-232F compared to rGD01-nsp6-232L (20.5 vs 26.7 puncta per cell, *P* = 0.048). A parallel approach using immunoblotting of LC3 showed an increase in LC3-II/ LC3-I ratio upon bafilomycin A1 (BafA1) treatment (which blocks the fusion of AP to AL) in rGD01-nsp6-232F at 24 hpi but no significant increase in the LC3-II/ LC3-I ratio in both viruses pair at 48 hpi (Fig. S3). Overall, the data reflect a minimal or an insignificant modulation of autophagy by the nsp6 L232F substitution.

To study the effect of zippering of ER, we generated an N-terminal GFP-tagged MERS-CoV nsp6 that can form punctate signals in the ER, as an indication of ER zippering. We followed approach from Ricciardi et al. (21) and confirmed only N-terminal-tagged MERS-CoV nsp6 will retain its native localization and zippering in ER (Fig. S4). The effect of ER zippering between nsp6232 Leu and Phe residue was compared by measuring the GFP puncta formation upon a drug-inducible expression in Hela cells. We observed no differences in GFP puncta formation at 1, 3, 6, and 8 hours post-induction, suggesting no significant impact of the nsp6 L232F substitution on ER zippering (Fig. 6C).

### MERS-CoV nsp6-232F showed higher virus egress through vesicles under electron microscopy

**Fig 7:**
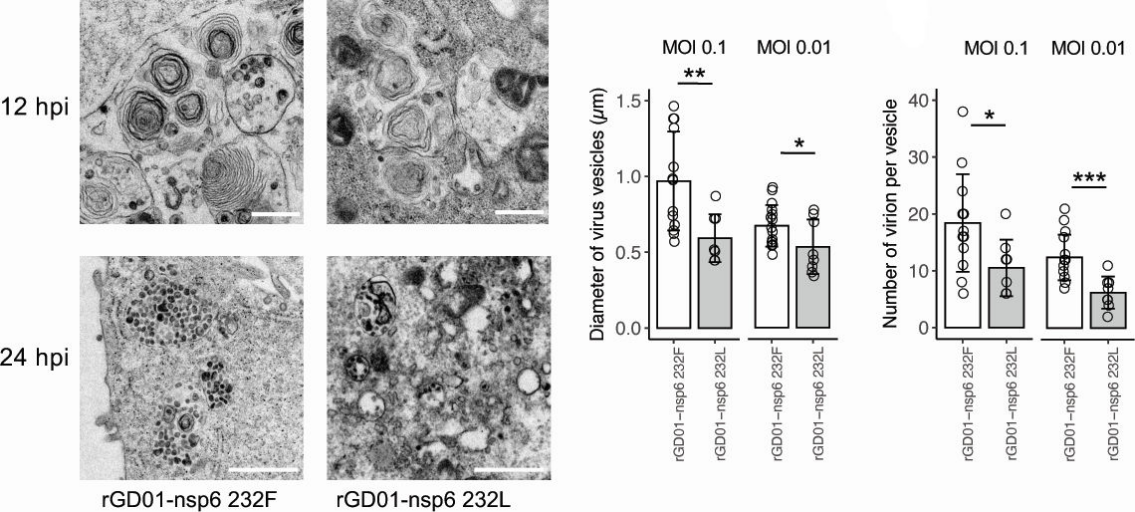
Nsp6 L232F substitution associated with higher virus egress through vesicles under electron microscopy. Electron microscopy of infected Vero cells at 12 hpi (MOI = 1) and at 24 hpi (MOI = 0.01). Diameters of virus vesicles and virion numbers per vesicle were measured from 24 hpi images using ImageJ. Number of cells analyzed: WT at MOI 0.1 (*n* = 13), at MOI 0.01 (*n* = 16); nsp6 232L at MOI 0.1 (*n* = 8), at MOI 0.01 (*n* = 8). Statistical tests were done using two-tailed Student’s *t* test: *P* < 0.05 (*); *P* < 0.01 (**); *P* < 0.001 (***). Scale bars = 500 nm.

Nsp6, together with nsp3 andnsp4, can induce DMV structures which are associated with the site of viral RNA replication (26). The quantification of DMV structures through EM may provide a mechanism for the increase in viral replication in nsp6 L232F substitution. Unfortunately, our attempts to quantify DMVs were not successful as the DMV structures were not well preserved in the EM images, possibly due to a prolonged formalin fixation in the virus inactivation step. However, our images showed virus-containing vesicles from infected Vero cell (Fig. 7). We observed a larger vesicle size and higher numbers of virus particles per vesicles in rGD01-nsp6-232F compared to rGD01-nsp6-232L-infected cells. Overall, these data support a higher viral production from nsp6 L232F mutant from a perspective of virus egress in the exocytic pathway.

## DISCUSSION

Inter-species transmission of viruses may be associated with the emergence of adaptive mutations. There have been repeated zoonotic introductions of MERS-CoV from camels to humans. In this study, we aimed to identify adaptive mutations of MERS-CoV. We identified nsp6 L232F substitution as the only one to occur preferentially in human sequences of clade B MERS-CoV, the virus genotype that shows current zoonotic transmission. This mutation emerged independently in multiple lineages (lineages 2, 3, 4 and 5) of human MERS-CoV without being present in the phylogenetically related camel viruses, evidence of convergent evolution in humans. So far, only one camel MERS-CoV has been shown to carry this L232F mutation (Genbank accession number: KT368870.1), and this was a clade B virus. Clade A MERS-CoV which only contains a few human sequences and clade C, which only contains camel sequences, all did not have the L232F mutation. We used next-generation sequencing (NGS) analysis to demonstrate that nsp6 F232 was not present as a minor mutant population in camel nasal swabs (Table S1). This suggests the mutation likely appeared after inter-species transmission to humans, although one cannot exclude the possibility that the mutation may be found rarely in viruses within camels.

Replication of isogenic virus pair (rGD01-nsp6-232F/rGD01-nsp6-232L) demonstrated that rGD01-nsp6-232F virus had significantly higher replication competence in *in vitro* and *ex vivo* cultures of human nasal and bronchial tissues. These data suggested that the nsp6 L232F may confer a higher intrinsic replication efficiency in the human upper and conducting airways. In hDPP4 knockin mice experimentally infected *in vivo* with these isogenic viruses, rGD01-nsp6-232F showed significantly higher viral titers in mouse lung. These results suggest an overall increase in virus production at an organism level is associated with nsp6-232F. Taken together, these findings may indicate that the nsp6 L232F substitution emerges in the human respiratory tract following zoonotic transmission as an adaptive mutation that confers replication advantage in the human respiratory tract. In the absence of a relevant experimental animal model of transmission, we cannot estimate the impact of this mutation on transmission between humans.

In order to define molecular mechanisms underlying the nsp6 L232F substitution phenotype, we studied innate immune antagonism, autophagy, and DMV formation. We found no significant difference in the expression of a number of type-I interferon downstream genes following infection with the pair of isogenic viruses in Calu-3 cells. In the hDPP4 knockin mouse, where we observed the higher viral titer in the mouse lung from rGD01-nsp6-232F infection, we found higher production of inflammatory cytokines such as IFNγ, MCP-1, and IP-10 (Fig. S5). But this may merely reflect a consequence of the higher viral replication observed with the rGD01-nsp6-232F virus rather than being a cause of this phenotype. Taken together, these data suggest the host innate immune responses were proportionate to the viral load, and nsp6 L232F may not alter innate immune responses.

MERS-CoV has been shown to restrict autophagy during infection. Ectopic expression of nsp6, Orf4b, and Orf5 individually can also lead to autophagy restriction (27). In this study, we only observed a subtle difference in autophagic restriction between nsp6 232L and 232F, indicating that it is unlikely to be explaining the viral replication difference. Similarly, nsp6 was recently demonstrated to play a role in DMV formation through ER zippering. We compared the zippering activity between GD01-nsp6-wt and GD01-nsp6-232L and identified no significant differences between them. In comparison, an nsp6 ΔSGF106-108 mutant observed in Alpha, Beta, Gamma, Eta, Iota, and Lambda variants of SARS-CoV-2 showed a higher ER-zippering activity (21). Additional studies using recombinant replicons showed an nsp6 ΔLSG105-107 mutant contributed reduced replication in Omicron against Delta variants of SARS-CoV-2, and using fluorescently labeled chimeric viruses showed Omicron spike, and nsp6 determines replication *in vitro* and pathogenicity in mice (28, 29). The nsp6 L232F in this study locates at the c-terminal cytoplasmic tail of the nsp6, while the ΔSGF 106–108 or ΔLSG105–107 in SARS-CoV-2 nsp6 is located in the luminal loop domain (25). The position difference could be an explanation to the observed phenotype. It will be of interest to study the effect of nsp6 L232F on RNA production using replicon systems. Our intention to explore DMV formation as a mechanism for mutation phenotype was not fruitful, largely due to the use of formalin as a fixative during virus inactivation necessitated by handling a bio-safety level 3 pathogen. The use of paraformaldehyde- and glutaraldehyde-based fixatives could better preserve DMV structures as performed in other studies using cryo-EM imaging (30), but our bio-safety protocols for MERS-CoV preclude much flexibility in this regard.

There are some limitations to this study. First, we did not address the impact of the nsp6 L232F substitution on disease pathogenicity. Although there are mouse models that manifest lethality upon MERS-CoV infection, each has limitations and is not ideal to study the pathogenicity of a putative nsp6 L232F substitution effect. Introducing the nsp6 L232F substitution in mouse-adapted strains which are pathogenic in mice is unlikely to be physiologically relevant. Second, we do not have an example where the nsp6 L232F substitution emerged within an individual. This would require serial sampling and sequencing of MERS-CoV viruses from very early in the zoonotic infection, and such data are not available. Furthermore, the first recognized zoonotic infection is often not the first zoonotic event in the chain of transmission from camels. Our findings provide a reason to initiate such systematic studies in zoonotic infections. The nsp6 L232F substitution was present in all viruses (*n* = 34) associated with the outbreak of MERS-CoV in South Korea in 2015, including in the index case of that case cluster (31–34). The index case likely acquired virus infection through travel in the Arabian Peninsula, and it is not clear if he was infected directly from a camel or if he acquired infection from another infected person. It is worth noting that multiple generations of human MERS transmission did not result in reversal of F232 to L232 residue in the South Korean outbreak. Another analysis of the MERS-CoV sequences from a hospital outbreak in Saudi Arabia (2018, *n* = 18) showed the same leucine genotype at the nsp6 232 position (35). This suggests that the nsp6 L232F substitution is not an indispensable human-adaptive mutation, and its effect on transmissibility remains uncertain. However, the phylogenetic evidence provides strong indirect evidence that this adaptation is occurring repeatedly in human infections. Thirdly, we did not examine the impact of the nsp6 L232F substitution on viral fitness in camels. Finally, we have not defined the mechanistic explanation for the replication advantage observed with this adaptive mutation.

In summary, our study demonstrated that amino acid substitution L232F in non-structural protein 6 is a likely human-adaptive mutation in clade B MERS coronaviruses. This amino acid substitution leads to higher replication competence in the human respiratory tract. These findings very likely show that MERS-CoV is repeatedly undergoing adaptive mutations and highlight the need for continuous surveillance in camels and humans to identify potential genotype changes relevant to human adaptation of MERS-CoV.

## MATERIALS AND METHODS

### Cells

Vero cells (ATCC CCL-81) and Hela cells (ATCC CCL-2) were maintained in Dulbecco’s modified Eagle medium (DMEM), supplemented with 10% fetal bovine serum (FBS), 25 mM HEPES, and 1% penicillin with streptomycin at 37°C, 5% CO_2_. Calu-3 cells (ATCC HTB-55) were maintained as above with the addition of 1% non-essential amino acid in the growth medium.

### Sequence-based analysis of the mutation

Previously published MERS-CoV sequences (full or partial genomes >10 kb) were retrieved from NCBI GenBank. Preliminary data cleaning was done by excluding sequences annotated as cell culture specimen and multiple sequences sampled from the same individual or sequences from clade A (no camel sequences) and C (no human sequences). Sequences were then aligned by MAFFT (https://mafft.cbrc.jp/alignment/server/index.html) and separated into either camel or human host. Pairwise genetic distances for each sequence in both data sets were calculated by the *p*-distance using SSE (v1.4) (36). A diversity threshold used for removing closely sampled human MERS-CoV was estimated based on the genetic diversity observed in the South Korea outbreak. We expect the South Korea outbreak originated from a single returning traveler will be a robust example to evaluate MERS-CoV evolution in humans only, without any involvement of camel hosts. Mean maximum *p*-distance of each sequence within the outbreak was calculated using SSE. We employed a pairwise *p*-distance of 3e−4, which is above the diversity for the South Korea outbreak (2.04e−4), as the cutoff threshold (Fig. 1B). Using this cutoff, 78 human MERS-CoVs with minimum pairwise *p*-distance >3e−4 were included for analysis, whereas for each cluster of highly similar sequences under the cutoff, only the sequence with the earliest collection date was retained for further analysis. Sequence pairs with a collection date difference longer than the transmission duration of the South Korea outbreak were also included. A total of 121 human sequences were included for further analysis. The same threshold and trimming procedures were applied to camel sequences, resulting in a total of 160 camel sequences. The combined 281 human and camel sequences formed the adjusted data set. The original 609 sequences were the unadjusted data set. For mutation counting, fasta files encoding the amino acid sequence of each ORF for human and camel data set were generated separately. Counting was implemented using R scripts. Statistical tests of independence of each mutation rate between human and camel hosts were performed using Fisher’s exact test with Bonferroni adjustment for multiple testing. Data sets are available in supporting information (Data Set S1).

### Phylogeny-based analysis of the mutation

To identify any mutation traits showed a dependent evolution with host species traits using phylogenetic information, we used BayesTraits to perform analysis by inputting samples of phylogenetic trees, and a metadata file denoted the binary mutation trait for each sequence (22). Analyses were done in both the adjusted and unadjusted data set. To prepare the samples of phylogenetic trees, human and camel sequences were aligned together and separated into two files encoding non-recombinant regions (1–16173, 24192–end, and 16174–24191) because a previous study found significant recombinant history in MERS-CoV genomes (37). Each non-recombinant alignment was inferred for a Bayesian phylogeny using MrBayes (38). At least 2 million Markov chain Monte Carlo generations were ran for each alignment with all parameters of the run having >100 effective sample sizes. Each file was run twice for analysis. A total of 500 random trees were retrieved as tree samples for BayesTraits analysis. To run BayesTraits, trees and metadata file were ran for discrete independent and dependent models with priors to an exponential with a mean of 10 and use the stepping stone sampler with 100 stones and 10,000 iterations per stone to estimate the marginal likelihood. A log Bayes factor of the marginal likelihood of each model was calculated for each mutation trait to evaluate the evidence of host-dependent evolution of the tested mutation, with the equation of: log Bayes factor = 2× (model likelihood of host-dependent evolution − model likelihood of host-independent evolution).

Epistatic interaction of mutations was evaluated by calculating the phi coefficient of mutation pairs in binary variables. A dataframe of sequences with binary mutation traits was used to calculate phi coefficient using R.

### Reverse genetics and rescue of recombinant viruses

The bacterial artificial chromosome plasmid (pBAC) construct of infectious MERS-CoV/China01(GD01) strain (GenBank accession no. KT006149.2) was kindly provided by Prof J. Zhao (Guangzhou Medical School, China). MERS-CoV GD01 contains nsp6 L232F. An independent risk assessment of the genetic modification of MERS-CoV in this study was implemented in the Safety Office, the University of Hong Kong. The experiment envisaged is a loss-of-function mutation as we start from a human virus possessing the putative “human-adaptive mutation” to mutate nsp232 to the camel genotype. To generate a recombinant GD01-nsp6 232L, a point mutation was introduced into the pBAC using a strategy of PCR mutagenesis and Gibson assembly. Primers were designed to amplify the whole genome of pBAC in long amplicon fragments of a size of 3–9 kb pairs each with 50-base pair overlapping regions. Specific forward and reverse primers containing the mutation were designed and used to amplify mutation carrying amplicons from the pBAC using PrimeSTAR Max DNA Polymerase (Takara). Each amplicon was gel purified and assembled into a circular form using Gibson Assembly reaction mix (New England Biolabs) according to the manufacturer’s protocol. The ligated reaction mix was then transformed into MegaX DH10B T1R electrocompTM cells (Thermo Fisher) using electroporation. Transformed cells were then recovered and screened for positive clones by antibiotic selection. Positive clones were screened by a PCR reaction and sub-cultured for maxi-prep using NucleoBond Xtra Maxi EF Kit (Macherey-Nagel) according to the kit instruction. Primers used are listed in the supporting information (Protocol S1).

Rescue of both viruses was carried by transfection of pBAC infectious clones into Vero cells using Lipofectamine 2000 (Thermo Fisher). Six hours post-transfection, the transfection medium was removed and supplemented with 2% FBS-supplemented DMEM. Success of virus rescue was confirmed by the observation of cytopathic effect 72 hours post-transfection. Rescued viruses were plaque purified in Vero cells and further sub-cultured to generate virus stocks, which were aliquoted and stored in −80°C until experimental use. Genetic identity of the virus stocks was confirmed by NGS. Titer of each stock of virus was determined by plaque assay.

### Replication kinetics *in vitro*

Calu-3 or Vero cells were seeded in 24-well tissue culture plates at 1.5 × 10^5^ cells per well in 10% FBS-supplemented DMEM. Prior to infection, cells were washed with PBS, and serum-free medium was added to the cells. Infection of MERS-CoV was done at a multiplicity of infection of 0.01 or 2 as indicated. After 1 hour of virus adsorption at 37°C, the virus inoculum was removed, and cells washed with PBS twice to remove the remaining virus inoculum. Wells were re-filled with 2% FBS-supplemented DMEM and incubated. Viral titers in the culture supernatants were quantified by the TCID_50_ assay. All experiments were done with three biological replicates.

### Growth competition assay

Vero cells were seeded with 1 × 10^6^ cells per well in a 6-well tissue culture plate. Ratios of rGD01-nsp6-232F vs rGD01-nsp6-232L at 9:1, 1:1, and 1:9 were prepared and added to cells at MOI 0.01. After 72 hours of incubation at 37°C, virus medium was harvested for viral RNA extraction and aliquoted for subsequent passage. For subsequent viral passage, 10 uL of virus medium was added to new plates seeded with cells supplemented in 2 mL of 2% FBS-supplemented DMEM and incubated another 72 hours of 37°C incubation. The procedures were repeated until passage 3. Viral RNA was extracted and sequenced across the mutation site by Sanger sequencing.

### *Ex vivo* cultures of nasal, bronchia, and lung tissues

Nasal, lung, and bronchial tissues from healthy parts of lung removed as part of routine surgical care, excess to diagnostic requirements, were used for these studies. These experiments were approved by the Institutional Review Board of the University of Hong Kong/Hospital Authority Hong Kong West Cluster (UW-13-104 and UW-20-862). Tissues from at least three separate donors were separately infected with each virus by incubating the tissue fragments in 1 mL of each virus at a dose of 10^6^ TCID_50_/mL for 1 hour at 33°C for nasal tissues and at 37°C for bronchus and lung tissues. The tissue fragments were then washed three times with PBS and replenished with cell culture medium DMEM and incubated further for 72 hours. Culture supernatants were harvested at the timepoints as indicated and quantitated for quantification of live virus using a TCID_50_ assay. The methods have been previously described in detail (39).

### Experimental infection of human DPP4 knockin mice

Human DPP4 knockin C57BL/6 mice 6–8 weeks old (kindly provided by Prof. Stanley Perlman, University of Iowa) were anesthetized and infected intranasally with 10e^4^ pfu of wild-type or mutant virus in 25 µL of PBS. Mock control mice were similarly treated with PBS only. Four mice from each virus group were euthanized at 4 hours, day 1, day 2, day 3, and day 5 post infection. The mouse lungs were removed and homogenized in 1 mL PBS solution and stored at −80°C until use. Supernatants of centrifuged homogenate were tested for viral titers for TCID_50_ assay in Vero cells. Ethics of animal experiments were approved by the university (CULATR: 5138-19).

### qPCR of innate immune gene expression

Virus-infected cells were washed gently with 1 mL of PBS twice. Cellular RNA was extracted and reverse transcribed into cDNA using random hexamers and measured for gene expression using qPCR assay. Primers used are listed in the supplementary information.

### Autophagic flux assay and LC3 immunoblotting

The effect of MERS-CoV infection on autophagic flux was determined by the transfection of a pHR′-CMV-eGFP-mCherry-LC3 plasmid (provided by Dr. Sumana Sanyal, University of Oxford) encoding fluorescent LC3, which emits as yellow puncta in autophagosomes and as red puncta in autolysosomes. Chloroquine (Sigma-Aldrich, C6628) and Hanks’ balanced salt solution (Thermo Fisher) were used as reduced and increased autophagic flux controls, respectively. Vero cells were transfected with pHR′-CMV-eGFP-mCherry-LC3 using Lipofectamine 2000, followed by MERS-CoV infection at MOI 0.01. Cells were fixed 24 hours post infection by 4% PFA and processed for confocal imaging.

### Electron microscopy

MERS-CoV-infected Vero cells were harvested at 9 hpi (MOI = 1) and 24 hpi (MOI = 0.01). Cells were washed once with PBS and scrapped from well plate in 10% formalin. Cells were centrifuged down into a pellet and fixed overnight. In the next day, cells were fixed in 2.5% glutaraldehyde in cacodylate buffer (0.1 M sodium cacodylate-HCl buffer pH 7.4) overnight, followed by processing, sectioning, and staining for transmission electron microscopy using Philips CM100.

### Plasmid construct for immunofluorescence study

Nsp6 gene fragments (WT and nsp6 232L) were amplified from the pair of recombinant pBAC-GD01 and cloned into a pCAGGS vector containing a FLAG tag either at the N-terminal or C-terminal, or without any tag using Gibson Assembly system. Inducible vectors were generated by cloning the nsp6 gene fragment into a doxycycline-inducible pCW vector containing a GFP tag at the N-terminal. Empty pCW was kindly provided by Alessia Ciarrocchi and Gloria Manzotti (Addgene plasmid # 184708). ER reporter protein GFP-cb5 was constructed by amplifying GFP with oligos containing the cytochrome b5 transmembrane domain sequence (5′-ITTVESNSSWWTNWVIPAISALVVALMYRLYMAED-3′) and cloned back into the pCAGGS vector using Gibson Assembly system.

### ER-zippering activity

Hela cells seeded on a cover glass were transfected with pCW-GFP-nsp6 or pCW-GFP-nsp6-232L using TransIT-LT1, according to the manufacturer’s protocol. At 24 hours post transfection, expression of nsp6 was induced by adding 1 µg mL^−1^ doxycycline (Sigma-Aldrich). Cells were fixed at 1, 3, 6, and 8 hours post induction by 4% paraformaldehyde and analyzed by confocal microscopy.

### Confocal microscopy and image analyses

Cells were imaged on a Zeiss LSM980 system. Images from the experiment were taken under the same laser parameter settings and magnification. Puncta from the images were counted and measured using Fiji software (ImageJ, National Institutes of Health) under the same color threshold settings across images.

### Statistical analysis

Comparisons were performed using two-sided unpaired Student’s *t*-test. All the data using *ex vivo* tissue cultures were compared using paired *t*-test. *P* values of <0.05 were considered statistically significant.

## Data Availability

All genomic sequence data are publicly available through NCBI GenBank. Accession numbers of viral sequences used in the phylogenetic analysis are listed in the supplemental information. NGS data of this study are available in the Sequence Read Archive (SRP467531). Source data are provided at Github (https://github.com/RScode23/nsp6). Correspondence and requests for materials should be addressed to Malik Peiris.

## SUPPLEMENTAL MATERIAL

Protocol S1 jvi.01369-23-s0001.xlsx Primers used in this study.

Supplemental figures jvi.01369-23-s0002.pdf Figures S1 to S6.

Table S1 jvi.01369-23-s0003.xlsx NGS analysis of minor variants at the codon position 232 of nsp6 in camel MERS-CoV isolates.

Data Set S1 part I jvi.01369-23-s0004.FASTA FASTA file of the 281 adjusted MERS-CoV sequences.

Data Set S1 part II jvi.01369-23-s0005.FASTA FASTA file of the 292 unadjusted human MERS-CoV sequences.

Data Set S1 part III jvi.01369-23-s0006.FASTA FASTA file of the 317 unadjusted camel MERS-CoV sequences.

ASM does not own the copyrights to Supplemental Material that may be linked to, or accessed through, an article. The authors have granted ASM a non-exclusive, world-wide license to publish the Supplemental Material files. Please contact the corresponding author directly for reuse.

## References

[1] Zaki AM, van Boheemen S, Bestebroer TM, Osterhaus A, Fouchier RAM. 2012. Isolation of a novel coronavirus from a man with pneumonia in Saudi Arabia. N Engl J Med 367:1814–1820. doi:10.1056/NEJMoa1211721 23075143

[2] WHO. 2016. Prioritizing diseases for research and development in emergency contexts. Available from: https://www.who.int/activities/prioritizing-diseases-for-research-and-development-in-emergency-contexts

[3] Mehand MS, Al-Shorbaji F, Millett P, Murgue B. 2018. The WHO R&D blueprint: 2018 review of emerging infectious diseases requiring urgent research and development efforts. Antiviral Res 159:63–67. doi:10.1016/j.antiviral.2018.09.009 30261226 PMC7113760

[4] Peiris M, Perlman S. 2022. Unresolved questions in the zoonotic transmission of MERS. Curr Opin Virol 52:258–264. doi:10.1016/j.coviro.2021.12.013 34999369 PMC8734234

[5] WHO. 2022. MERS situation update. Available from: https://www.emro.who.int/health-topics/mers-cov/mers-outbreaks.html

[6] Haagmans BL, Al Dhahiry SHS, Reusken C, Raj VS, Galiano M, Myers R, Godeke G-J, Jonges M, Farag E, Diab A, Ghobashy H, Alhajri F, Al-Thani M, Al-Marri SA, Al Romaihi HE, Al Khal A, Bermingham A, Osterhaus A, AlHajri MM, Koopmans MPG. 2014. Middle East respiratory syndrome coronavirus in dromedary camels: an outbreak investigation. Lancet Infect Dis 14:140–145. doi:10.1016/S1473-3099(13)70690-X 24355866 PMC7106553

[7] Madani TA, Azhar EI, Hashem AM. 2014. Evidence for camel-to-human transmission of MERS coronavirus. N Engl J Med 371:1360. doi:10.1056/NEJMc1409847 25271614

[8] Memish ZA, Perlman S, Van Kerkhove MD, Zumla A. 2020. Middle East respiratory syndrome. Lancet 395:1063–1077. doi:10.1016/S0140-6736(19)33221-0 32145185 PMC7155742

[9] Assiri A, McGeer A, Perl TM, Price CS, Al Rabeeah AA, Cummings DAT, Alabdullatif ZN, Assad M, Almulhim A, Makhdoom H, Madani H, Alhakeem R, Al-Tawfiq JA, Cotten M, Watson SJ, Kellam P, Zumla AI, Memish ZA, KSA MERS-CoV Investigation Team. 2013. Hospital outbreak of Middle East respiratory syndrome coronavirus. N Engl J Med 369:407–416. doi:10.1056/NEJMoa1306742 23782161 PMC4029105

[10] Al-Abdallat MM, Payne DC, Alqasrawi S, Rha B, Tohme RA, Abedi GR, Al Nsour M, Iblan I, Jarour N, Farag NH, Haddadin A, Al-Sanouri T, Tamin A, Harcourt JL, Kuhar DT, Swerdlow DL, Erdman DD, Pallansch MA, Haynes LM, Gerber SI, Jordan MERS-CoV Investigation Team. 2014. Hospital-associated outbreak of Middle East respiratory syndrome coronavirus: a serologic, epidemiologic, and clinical description. Clin Infect Dis 59:1225–1233. doi:10.1093/cid/ciu359 24829216 PMC4834865

[11] Cho SY, Kang J-M, Ha YE, Park GE, Lee JY, Ko J-H, Lee JY, Kim JM, Kang C-I, Jo IJ, Ryu JG, Choi JR, Kim S, Huh HJ, Ki C-S, Kang E-S, Peck KR, Dhong H-J, Song J-H, Chung DR, Kim Y-J. 2016. MERS-CoV outbreak following a single patient exposure in an emergency room in South Korea: an epidemiological outbreak study. Lancet 388:994–1001. doi:10.1016/S0140-6736(16)30623-7 27402381 PMC7159268

[12] Payne DC, Biggs HM, Al-Abdallat MM, Alqasrawi S, Lu X, Abedi GR, Haddadin A, Iblan I, Alsanouri T, Al Nsour M, Sheikh Ali S, Rha B, Trivedi SU, Rasheed MAU, Tamin A, Lamers MM, Haagmans BL, Erdman DD, Thornburg NJ, Gerber SI. 2018. Multihospital outbreak of a Middle East respiratory syndrome coronavirus deletion variant, Jordan: a molecular, serologic, and epidemiologic investigation. Open Forum Infect Dis 5:fy095. doi:10.1093/ofid/ofy095 PMC5965092 30294616

[13] Chinese S. 2004. Molecular evolution of the SARS coronavirus during the course of the SARS epidemic in China. Science 303:1666–1669. doi:10.1126/science.1092002 14752165

[14] Subbarao EK, London W, Murphy BR. 1993. A single amino acid in the PB2 gene of influenza A virus is a determinant of host range. J Virol 67:1761–1764. doi:10.1128/JVI.67.4.1761-1764.1993 8445709 PMC240216

[15] Volz E, Hill V, McCrone JT, Price A, Jorgensen D, O’Toole Á, Southgate J, Johnson R, Jackson B, Nascimento FF, et al.. 2021. Evaluating the effects of SARS-CoV-2 spike mutation D614G on transmissibility and pathogenicity. Cell 184:64–75. doi:10.1016/j.cell.2020.11.020 33275900 PMC7674007

[16] Lamers MM, Raj VS, Shafei M, Ali SS, Abdallh SM, Gazo M, Nofal S, Lu X, Erdman DD, Koopmans MP, Abdallat M, Haddadin A, Haagmans BL. 2016. Deletion variants of Middle East respiratory syndrome coronavirus from humans, Jordan, 2015. Emerg Infect Dis 22:716–719. doi:10.3201/eid2204.152065 26981770 PMC4806954

[17] Xie Q, Cao Y, Su J, Wu J, Wu X, Wan C, He M, Ke C, Zhang B, Zhao W. 2017. Two deletion variants of Middle East respiratory syndrome coronavirus found in a patient with characteristic symptoms. Arch Virol 162:2445–2449. doi:10.1007/s00705-017-3361-x 28421366 PMC5506503

[18] Cottam EM, Whelband MC, Wileman T. 2014. Coronavirus NSP6 restricts autophagosome expansion. Autophagy 10:1426–1441. doi:10.4161/auto.29309 24991833 PMC4203519

[19] Nishitsuji H, Iwahori S, Ohmori M, Shimotohno K, Murata T. 2022. Ubiquitination of SARS-CoV-2 nsp6 and ORF7a facilitates NF-κB activation. mBio 13:e0097122. doi:10.1128/mbio.00971-22 35856559 PMC9426613

[20] Xia H, Cao Z, Xie X, Zhang X, Chen J-C, Wang H, Menachery VD, Rajsbaum R, Shi P-Y. 2020. Evasion of type I interferon by SARS-CoV-2. Cell Rep 33:108234. doi:10.1016/j.celrep.2020.108234 32979938 PMC7501843

[21] Ricciardi S, Guarino AM, Giaquinto L, Polishchuk EV, Santoro M, Di Tullio G, Wilson C, Panariello F, Soares VC, Dias SSG, Santos JC, Souza TML, Fusco G, Viscardi M, Brandi S, Bozza PT, Polishchuk RS, Venditti R, De Matteis MA. 2022. The role of nsp6 in the biogenesis of the SARS-CoV-2 replication organelle. Nature 606:761–768. doi:10.1038/s41586-022-04835-6 35551511 PMC7612910

[22] Pagel M, Meade A, Barker D, Thorne J. 2004. Bayesian estimation of ancestral character states on phylogenies. Syst Biol 53:673–684. doi:10.1080/10635150490522232 15545248

[23] Li K, Wohlford-Lenane CL, Channappanavar R, Park J-E, Earnest JT, Bair TB, Bates AM, Brogden KA, Flaherty HA, Gallagher T, Meyerholz DK, Perlman S, McCray PB. 2017. Mouse-adapted MERS coronavirus causes lethal lung disease in human DPP4 knockin mice. Proc Natl Acad Sci U S A 114:E3119–E3128. doi:10.1073/pnas.1619109114 28348219 PMC5393213

[24] Baliji S, Cammer SA, Sobral B, Baker SC. 2009. Detection of nonstructural protein 6 in murine coronavirus-infected cells and analysis of the transmembrane topology by using bioinformatics and molecular approaches. J Virol 83:6957–6962. doi:10.1128/JVI.00254-09 19386712 PMC2698535

[25] Feng S, O’Brien A, Chen D-Y, Saeed M, Baker SC. 2023. SARS-CoV-2 nonstructural protein 6 from alpha to omicron: evolution of a transmembrane protein. mBio 14:e0068823. doi:10.1128/mbio.00688-23 37477426 PMC10470488

[26] Angelini MM, Akhlaghpour M, Neuman BW, Buchmeier MJ. 2013. Severe acute respiratory syndrome coronavirus nonstructural proteins 3, 4, and 6 induce double-membrane vesicles. mBio 4:e00524-13. doi:10.1128/mBio.00524-13 PMC3747587 23943763

[27] Gassen NC, Niemeyer D, Muth D, Corman VM, Martinelli S, Gassen A, Hafner K, Papies J, Mösbauer K, Zellner A, Zannas AS, Herrmann A, Holsboer F, Brack-Werner R, Boshart M, Müller-Myhsok B, Drosten C, Müller MA, Rein T. 2019. SKP2 attenuates autophagy through beclin1-ubiquitination and its inhibition reduces MERS-coronavirus infection. Nat Commun 10:5770. doi:10.1038/s41467-019-13659-4 31852899 PMC6920372

[28] Taha TY, Chen IP, Hayashi JM, Tabata T, Walcott K, Kimmerly GR, Syed AM, Ciling A, Suryawanshi RK, Martin HS, Bach BH, Tsou C-L, Montano M, Khalid MM, Sreekumar BK, Renuka Kumar G, Wyman S, Doudna JA, Ott M. 2023. Rapid assembly of SARS-CoV-2 genomes reveals attenuation of the omicron BA.1 variant through nsp6. Nat Commun 14:2308. doi:10.1038/s41467-023-37787-0 37085489 PMC10120482

[29] Chen D-Y, Chin CV, Kenney D, Tavares AH, Khan N, Conway HL, Liu G, Choudhary MC, Gertje HP, O’Connell AK, Adams S, Kotton DN, Herrmann A, Ensser A, Connor JH, Bosmann M, Li JZ, Gack MU, Baker SC, Kirchdoerfer RN, Kataria Y, Crossland NA, Douam F, Saeed M. 2023. Spike and nsp6 are key determinants of SARS-CoV-2 omicron BA.1 attenuation. Nature 615:143–150. doi:10.1038/s41586-023-05697-2 36630998

[30] Klein S, Cortese M, Winter SL, Wachsmuth-Melm M, Neufeldt CJ, Cerikan B, Stanifer ML, Boulant S, Bartenschlager R, Chlanda P. 2020. SARS-CoV-2 structure and replication characterized by in situ cryo-electron tomography. Nat Commun 11:5885. doi:10.1038/s41467-020-19619-7 33208793 PMC7676268

[31] Lee JY, Kim Y-J, Chung EH, Kim D-W, Jeong I, Kim Y, Yun M-R, Kim SS, Kim G, Joh J-S. 2017. The clinical and virological features of the first imported case causing MERS-CoV outbreak in South Korea, 2015. BMC Infect Dis 17:498. doi:10.1186/s12879-017-2576-5 28709419 PMC5512736

[32] Seong M-W, Kim SY, Corman VM, Kim TS, Cho SI, Kim MJ, Lee SJ, Lee J-S, Seo SH, Ahn JS, Yu BS, Park N, Oh M, Park WB, Lee JY, Kim G, Joh JS, Jeong I, Kim EC, Drosten C, Park SS. 2016. Microevolution of outbreak-associated Middle East respiratory syndrome coronavirus, South Korea, 2015. Emerg Infect Dis 22:327–330. doi:10.3201/eid2202.151700 26814649 PMC4734539

[33] Kim Y-J, Cho Y-J, Kim D-W, Yang J-S, Kim H, Park S, Han YW, Yun M-R, Lee HS, Kim A-R, Heo DR, Kim JA, Kim SJ, Jung H-D, Kim N, Yoon S-H, Nam J-G, Kang HJ, Cheong H-M, Lee J-S, Chun J, Kim SS. 2015. Complete genome sequence of Middle East respiratory syndrome coronavirus KOR/KNIH/002_05_2015, isolated in South Korea. Genome Announc 3:e00787-15. doi:10.1128/genomeA.00787-15 26272558 PMC4536669

[34] Kim Y, Cheon S, Min C-K, Sohn KM, Kang YJ, Cha Y-J, Kang J-I, Han SK, Ha N-Y, Kim G, Aigerim A, Shin HM, Choi M-S, Kim S, Cho H-S, Kim Y-S, Cho N-H. 2016. Spread of mutant Middle East respiratory syndrome coronavirus with reduced affinity to human CD26 during the South Korean outbreak. mBio 7:e00019. doi:10.1128/mBio.00019-16 26933050 PMC4810480

[35] Barry M, Phan MV, Akkielah L, Al-Majed F, Alhetheel A, Somily A, Alsubaie SS, McNabb SJ, Cotten M, Zumla A, Memish ZA. 2020. Nosocomial outbreak of the Middle East respiratory syndrome coronavirus: a phylogenetic, epidemiological, clinical and infection control analysis. Travel Med Infect Dis 37:101807. doi:10.1016/j.tmaid.2020.101807 32599173 PMC7319941

[36] Simmonds P. 2012. SSE: a nucleotide and amino acid sequence analysis platform. BMC Res Notes 5:50. doi:10.1186/1756-0500-5-50 22264264 PMC3292810

[37] Sabir JSM, Lam T-Y, Ahmed MMM, Li L, Shen Y, Abo-Aba SEM, Qureshi MI, Abu-Zeid M, Zhang Y, Khiyami MA, Alharbi NS, Hajrah NH, Sabir MJ, Mutwakil MHZ, Kabli SA, Alsulaimany FAS, Obaid AY, Zhou B, Smith DK, Holmes EC, Zhu H, Guan Y. 2016. Co-circulation of three camel coronavirus species and recombination of MERS-CoVs in Saudi Arabia. Science 351:81–84. doi:10.1126/science.aac8608 26678874

[38] Ronquist F, Teslenko M, van der Mark P, Ayres DL, Darling A, Höhna S, Larget B, Liu L, Suchard MA, Huelsenbeck JP. 2012. MrBayes 3.2: efficient Bayesian phylogenetic inference and model choice across a large model space. Syst Biol 61:539–542. doi:10.1093/sysbio/sys029 22357727 PMC3329765

[39] Chan RWY, Hemida MG, Kayali G, Chu DKW, Poon LLM, Alnaeem A, Ali MA, Tao KP, Ng HY, Chan MCW, Guan Y, Nicholls JM, Peiris JSM. 2014. Tropism and replication of Middle East respiratory syndrome coronavirus from dromedary camels in the human respiratory tract: an in-vitro and ex-vivo study. Lancet Respir Med 2:813–822. doi:10.1016/S2213-2600(14)70158-4 25174549 PMC7164818

