# Supplementary material for: Amino acid substitution L232F in non-structural protein 6 identified as a possible human-adaptive mutation in clade B MERS coronaviruses
Source: J Virol. 2023 Dec 1;97(12):e01369-23. doi: 10.1128/jvi.01369-23 (PMC10734512; doi:10.1128/jvi.01369-23)
Supplement: Supplemental figures — Figures S1 to S6. [file jvi.01369-23-s0002.pdf]

Supplemental Materials

Figures

A

Taxon	nsp6 L232F (20/20)	nsp3 G128S (3/27)	nsp3 P246R (3/27)	nsp3 V522I (3/27)	nsp3 A609T (3/26)	nsp13 V241A (1/7)	ORF3 P86L (8/72)	ORF4b M6T (7/71)	M F123I (8/76)	N A300V (3/9)
KF600630	+									
KJ156910	+									
KJ156916	+									
KM027256	+									
KP209312	+									
KT121578	+									
KT121580	+									
KT326819	+						+		+	
KT806055	+									
KU710264	+									
KX154686	+						+	+	+	
KY689142	+									+
MG011341	+	+	+	+	+		+	+	+	
MG011362	+						+	+	+	
MG757593	+						+	+	+	
MH013216	+									+
MH306207	+					+	+	+	+	+
MK052676	+									
MK462248	+	+	+	+	+		+	+	+	
MN365232	+	+	+	+	+		+	+	+	

B

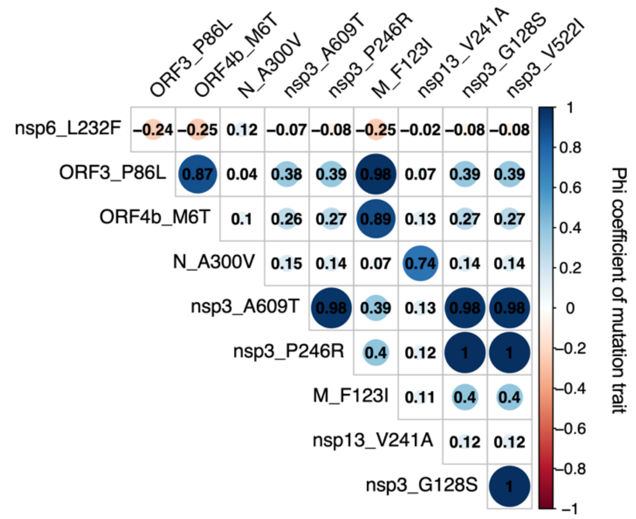

S1 Fig. Nsp6 L232F showed lack of epistasis with other mutations. (A) Mutation profile of the 20 MERS-CoV with nsp6 L232F. + indicates presence of the mutation. Numbers of mutations in the 20 sequences per total observed sequences were indicated. (B) Phi correlation coefficient of the top 10 mutation hit traits in binary. Phi coefficient of ±0-0.3 indicates little or no association; ±0.3-0.7 indicates weak association; ±0.7-1indicates strong association.

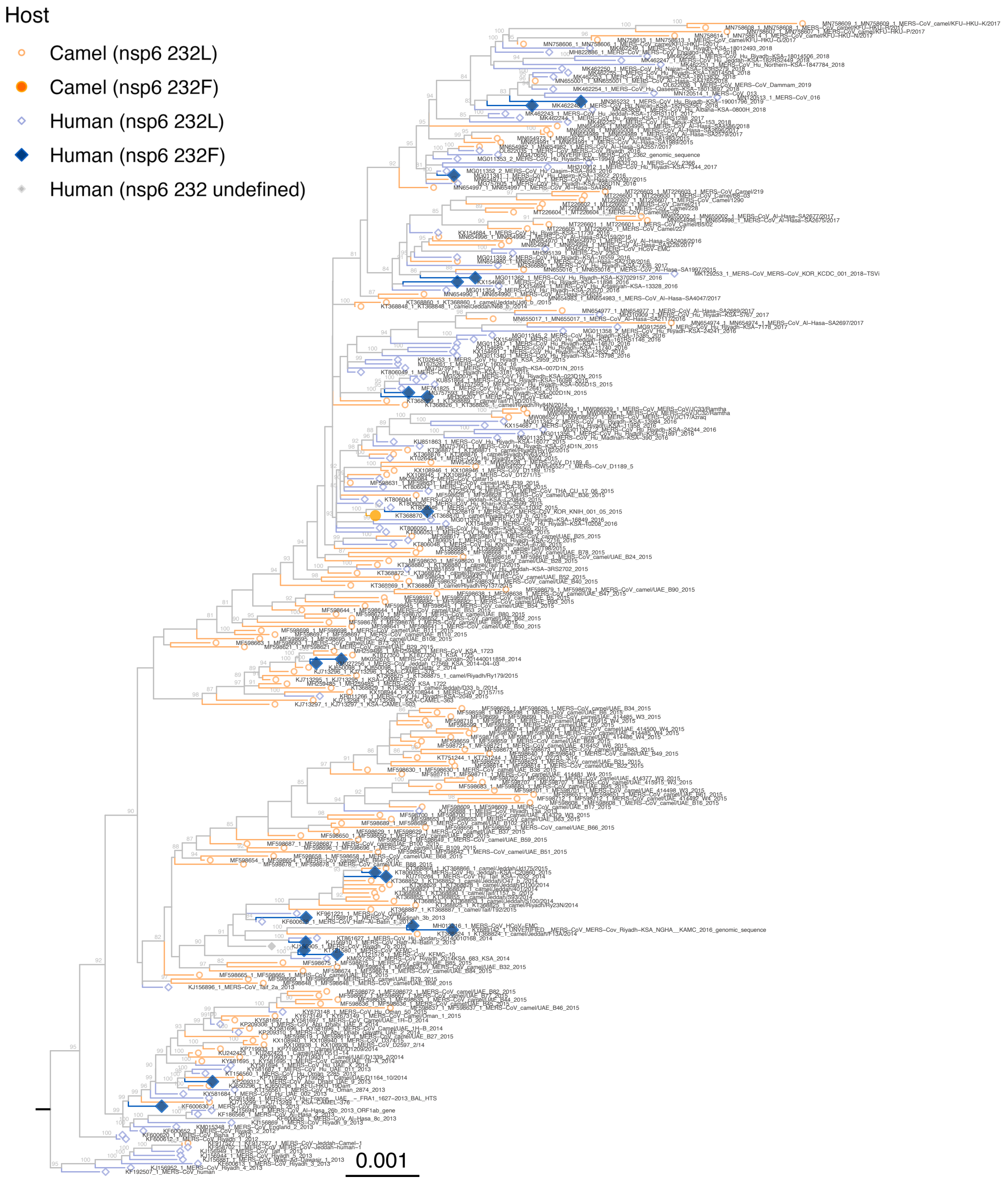

S2 Fig. PhyML tree of 281 MERS-CoV genomes from human and dromedary camel labelled with the nsp6 L232F substitution. Tree was built with IQ-Tree v1.6.12 using GTR+I+R substitution model. Yellow circles represent camel sequences; blue diamonds represent human sequences. Taxon with filled color shape indicates the presence of the nsp6 L232F substitution. Bootstrap supports of nodes were tested using non-parametric bootstrap test and indicated with values >80. Scale bar, 0.001 substitution per site.

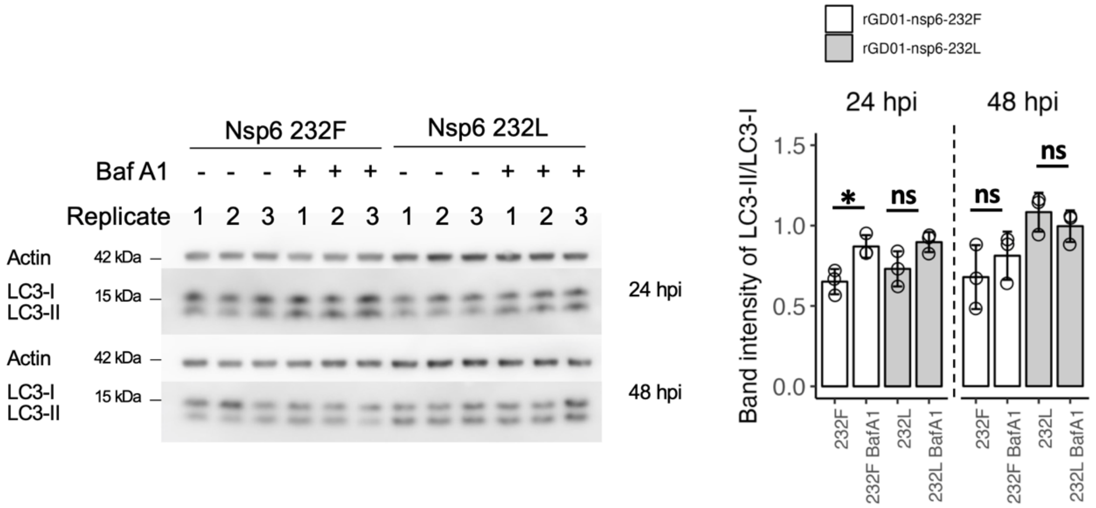

S3 Fig. Nsp6 L232F substitution showed minimal modulation in autophagic restriction. Western blot of LC3-I/II in Vero cells infected at MOI=0.01. Bafilomycin A1 (BafA1, 0.1 μM) was added to cells for 2 h before harvest for protein lysate. Cells were extracted for protein using RIPA buffer with freshly supplemented protease inhibitors. Protein samples were subsequently added with sample buffer and incubated at 95°C for 10 mins. Western blotting of LC3-B was labelled with anti-LC3-B primary antibodies (1:1000, Cell Signaling Technology, #3868) and was normalized with anti-Actin antibodies (1:4000, Thermo, MA1-744). Band intensities of LC3B-I and LC3B-II were measured in Fiji software (ImageJ, National Institutes of Health) and normalized to band intensity of Actin. Assays were performed in triplicates. Statistical tests were done using two tailed Student's t test: $p \geq 0.05$ (ns); $p < 0.05$ (*); $p < 0.01$ (**); $p < 0.001$ (***).

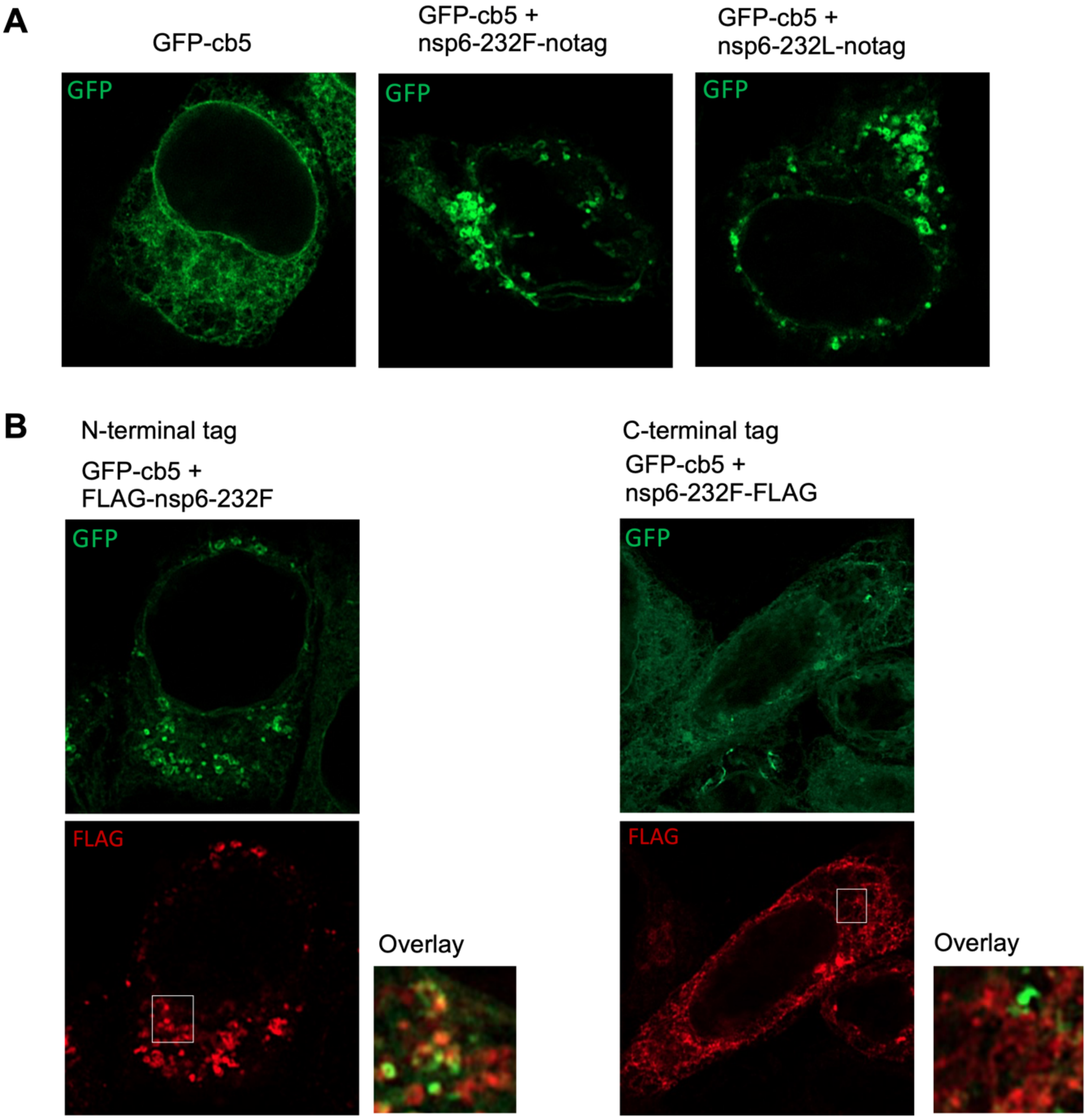

S4 Fig. MERS-CoV nsp6 expression induces ER zippering only when tagged at the N-terminal position. (A) Hela cells expressing an ER reporter protein using a recombinant GFP with a cytochrome b5 hydrophobic sequence (GFP-cb5) or co-expressing with nsp6-232F or nsp6-232L without tag. No GFP puncta observed in GFP-cb5 expression. GFP puncta observed GFP-cb5 co-expressed with nsp6-232F or nsp6-F232L, indicating zippered ER structures. (B) Hela cells co-expressing GFP-cb5 with either a N-terminal or C-terminal FLAG tagged nsp6 WT. Only N-terminal tagged nsp6 showed zippered ER structures. Insets, merged image of boxed areas. Hela cells were seeded on a cover glass placed in a 12 well plate. In the next day, cells were transfected with pCAGGS-nsp6, pCAGGS-FLAG-nsp6, pCAGGS-nsp6-FLAG, or pCAGGS-GFP-cb5 using TransIT-LT1 (Mirus Bio) according to the manufacturer's protocol. At 24h post transfection, cells were fixed in 4% paraformaldehyde, permeabilized, stained using a primary rabbit polyclonal anti-FLAG (1:100, Bethyl, A190-102A) and a secondary goat anti-rabbit IgG H&L (1:200, Alexa Fluor® 647).

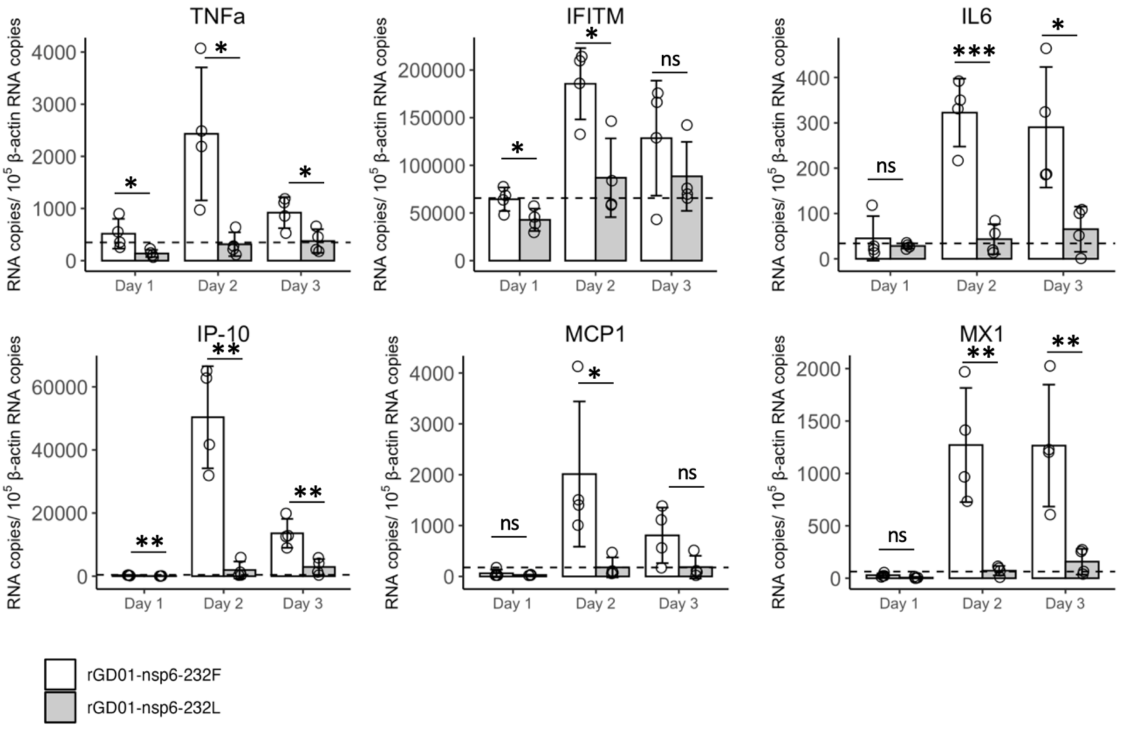

S5 Fig. Innate immune gene expression in the lungs of human DPP4 knockin C57BL/6 mice infected with the isogenic virus pair. RNA expression of immune genes were measured by qPCR. n=4 for each group. Horizontal dotted line represents expression level in mock controls. Statistical comparisons were performed using Student's t-test.

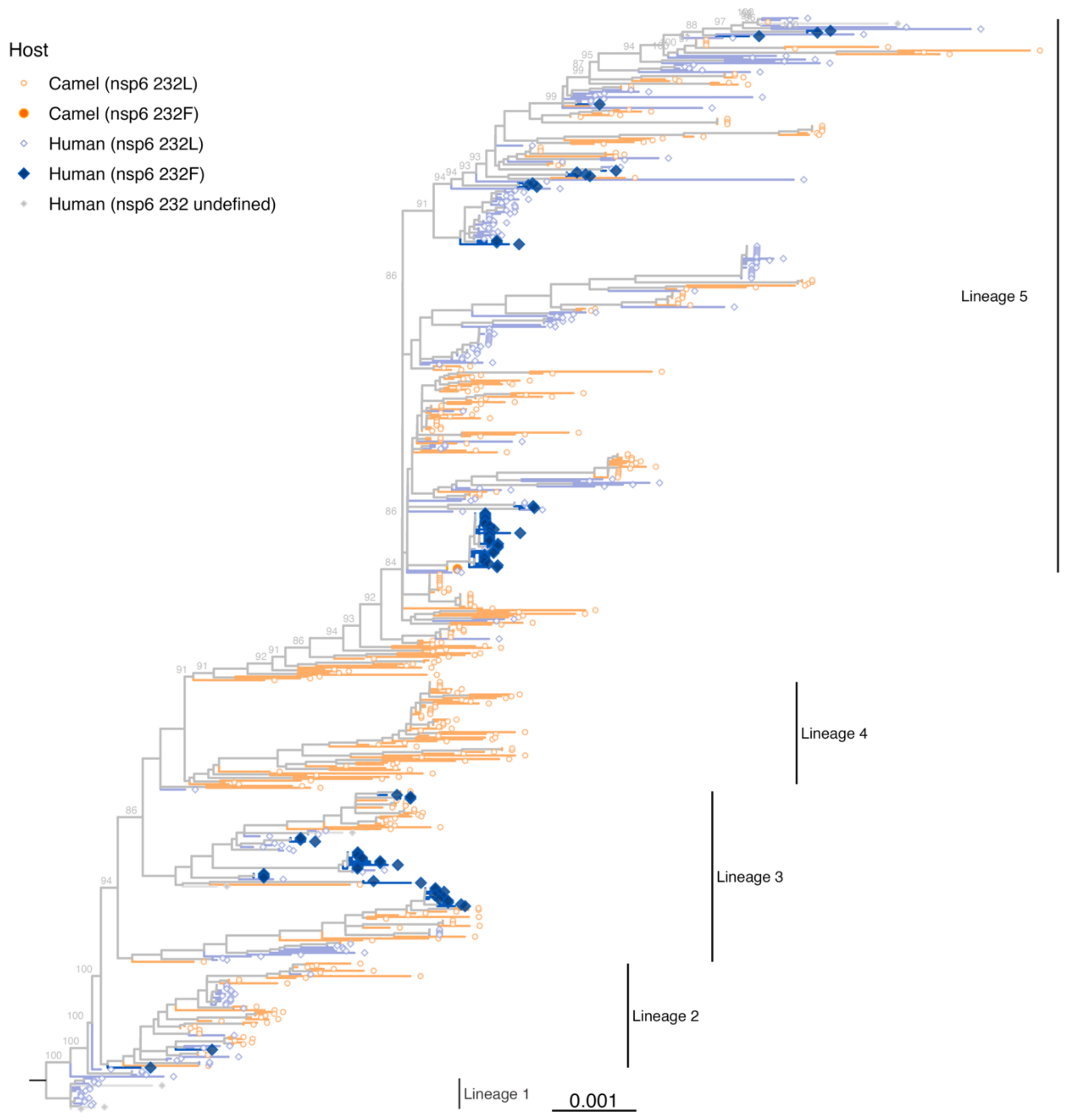

S6 Fig. PhyML tree of the unadjusted dataset consists of 609 MERS-CoV genomes from human and dromedary camel labelled with the nsp6 L232F substitution. Tree was built with IQ-Tree v1.6.12 using GTR+I+R substitution model. Yellow circles represent camel sequences; blue diamonds represent human sequences. Taxon with filled color shape indicates the presence of the nsp6 L232F substitution. Bootstrap supports of nodes were tested using non-parametric bootstrap test and indicated with values >80. Scale bar, 0.001 substitution per site.
